# Soil conditions and the plant microbiome boost the accumulation of monoterpenes in the fruit of *Citrus reticulata* ‘Chachi’

**DOI:** 10.1186/s40168-023-01504-2

**Published:** 2023-03-28

**Authors:** Jianmu Su, Yayu Wang, Mei Bai, Tianhua Peng, Huisi Li, Hui-Juan Xu, Guifang Guo, Haiyi Bai, Ning Rong, Sunil Kumar Sahu, Hanjun He, Xiangxiu Liang, Canzhi Jin, Wei Liu, Mikael Lenz Strube, Lone Gram, Yongtao Li, Ertao Wang, Huan Liu, Hong Wu

**Affiliations:** 1 State Key Laboratory for Conservation and Utilization of Subtropical Agro-Bioresources, Guangdong Laboratory for Lingnan Modern Agriculture, College of Life Sciences, South China Agricultural University, Guangzhou, 510642 China; 2 State Key Laboratory of Agricultural Genomics, BGI-Shenzhen, Shenzhen, 518083 China; 3 Department of Biotechnology and Biomedicine, Technical University of Denmark, Søltofts Plads, 2800 Kgs. Lyngby, Denmark; 4 Joint Institute for Environmental Research & Education, College of Natural Resources and Environment, South China Agricultural University, Guangzhou, 510642 China; 5 National Key Laboratory of Plant Molecular Genetics, Chinese Academy of Sciences Center for Excellence in Molecular Plant Sciences, Institute of Plant Physiology and Ecology, Shanghai Institutes for Biological Sciences, Chinese Academy of Sciences, Shanghai, 200032 China

**Keywords:** Soil conditions, Plant microbiome, Monoterpenes, *Citrus reticulata* ‘Chachi’, Citri Reticulatae Pericarpium

## Abstract

**Background:**

The medicinal material quality of *Citrus reticulata* ‘Chachi’ differs depending on the bioactive components influenced by the planting area. Environmental factors, such as soil nutrients, the plant-associated microbiome and climatic conditions, play important roles in the accumulation of bioactive components in citrus. However, how these environmental factors mediate the production of bioactive components of medicinal plants remains understudied.

**Results:**

Here, a multi-omics approach was used to clarify the role of environmental factors such as soil nutrients and the root-associated microbiome on the accumulation of monoterpenes in the peel of *C. reticulata* ‘Chachi’ procured from core (geo-authentic product region) and non-core (non-geo-authentic product region) geographical regions. The soil environment (high salinity, Mg, Mn and K) enhanced the monoterpene content by promoting the expression of salt stress-responsive genes and terpene backbone synthase in the host plants from the core region. The microbial effects on the monoterpene accumulation of citrus from the core region were further verified by synthetic community (SynCom) experiments. Rhizosphere microorganisms activated terpene synthesis and promoted monoterpene accumulation through interactions with the host immune system. Endophyte microorganisms derived from soil with the potential for terpene synthesis might enhance monoterpene accumulation in citrus by providing precursors of monoterpenes.

**Conclusions:**

Overall, this study demonstrated that both soil properties and the soil microbiome impacted monoterpene production in citrus peel, thus providing an essential basis for increasing fruit quality via reasonable fertilization and precision microbiota management.

Video Abstract

**Supplementary Information:**

The online version contains supplementary material available at 10.1186/s40168-023-01504-2.

## Background

The quality of medicinal plants is impacted by the genetic background and several other factors [1, 2], such as soil nutrients [3], the plant-associated microbiome [4] and climatic conditions [5]. Soil nutrients can directly impact the bioactive components of medicinal plants [6, 7] and influence the soil microbial composition [8, 9]. In recent years, the diversity and relative abundance of different taxonomic groups in the root microbiome of major crops, such as rice [10], corn [11, 12] and citrus [13], have been well documented. Recent studies have shown the importance of the soil microbiome in plant growth, health and disease and that it can promote the absorption of nutrients by roots [14], enhance plant resistance to diseases [15, 16] and insects [17] and improve resistance to drought [18], salt [19] and heavy metals [20]. Such studies enable our understanding of the impact of the microbiome on the plant immune response and other physiological processes.

In turn, plants employ a diversity of mechanisms to modulate their microbiome, including the exudation of secondary metabolites [21] and the coordinated action of different defence responses [22]. Indeed, there is increasing evidence that the structure of plant microbiomes is the result of a series of interactions between the plant, the microbes and their environmental physical and chemical conditions [23]. For example, terpenes and terpenoids, which can be produced by bacteria and plants, are important mediators of plant–soil microbiome interactions [24]. Root exudates such as triterpenes [25], benzoxazinoids [26] and organic carbons [27] are a vital driving force in the assembly of rhizosphere microbiomes. The rhizosphere is a critical interface supporting the exchange of resources between plants and their associated soil environment, which is actually a complex ecosystem hosting diverse bacteria [28]. However, little is known about the communication between interacting partners that impacts their phenotypic changes.

Citri Reticulatae Pericarpium (CRP), the dried ripe peel of *Citrus reticulata* Blanco or its cultivars, is not only consumed as a dietary supplement in China and other eastern countries but also used as one of the most popular traditional medicinal herbs in clinical practice for the treatment of indigestion and inflammatory syndromes of the respiratory tract [29]. *C. reticulata* ‘Chachi’ has been cultivated in Xinhui County of Guangdong Province (known as the core region) and is also popularly known as ‘Guangchenpi’ (GCRP), which has always been well regarded as the best national product with respect to geo-herbalism [30]. As the main raw material of GCRP, the peel of *C. reticulata* ‘Chachi’ is authoritatively authenticated as a genuine regional drug. Essential oils [31] and flavonoids [32] are among the most important bioactive components of *C. reticulata* ‘Chachi’, with a relatively high abundance. Terpenes, which are the main components of essential oils, have excellent antioxidant and antifungal activities [33]. Root microorganisms can affect the content of secondary metabolites of medicinal plants by affecting the synthesis of medicinal ingredients [34]. However, the relationship between the accumulation of bioactive components of *C. reticulata* ‘Chachi’ and the root-associated microbiome is still unclear.

In this study, multi-omics analyses were used to investigate the effects of soil conditions and microorganisms on the production of secondary metabolites in the peel of *C. reticulata* ‘Chachi’ from core and non-core regions. We substantiated the links between the content of monoterpenes, gene expression, soil properties and the root-associated microbiome. We verified the microbial effects on the production of monoterpenes in *C. reticulata* ‘Chachi’ using bacterial strains isolated from the field. We found that root microbes affected bioactive component accumulation in *C. reticulata* ‘Chachi’ in an immune-dependent manner.

## Results

### Comparative analysis of essential oils in the citrus peel of different planting regions

**Fig. 1:**
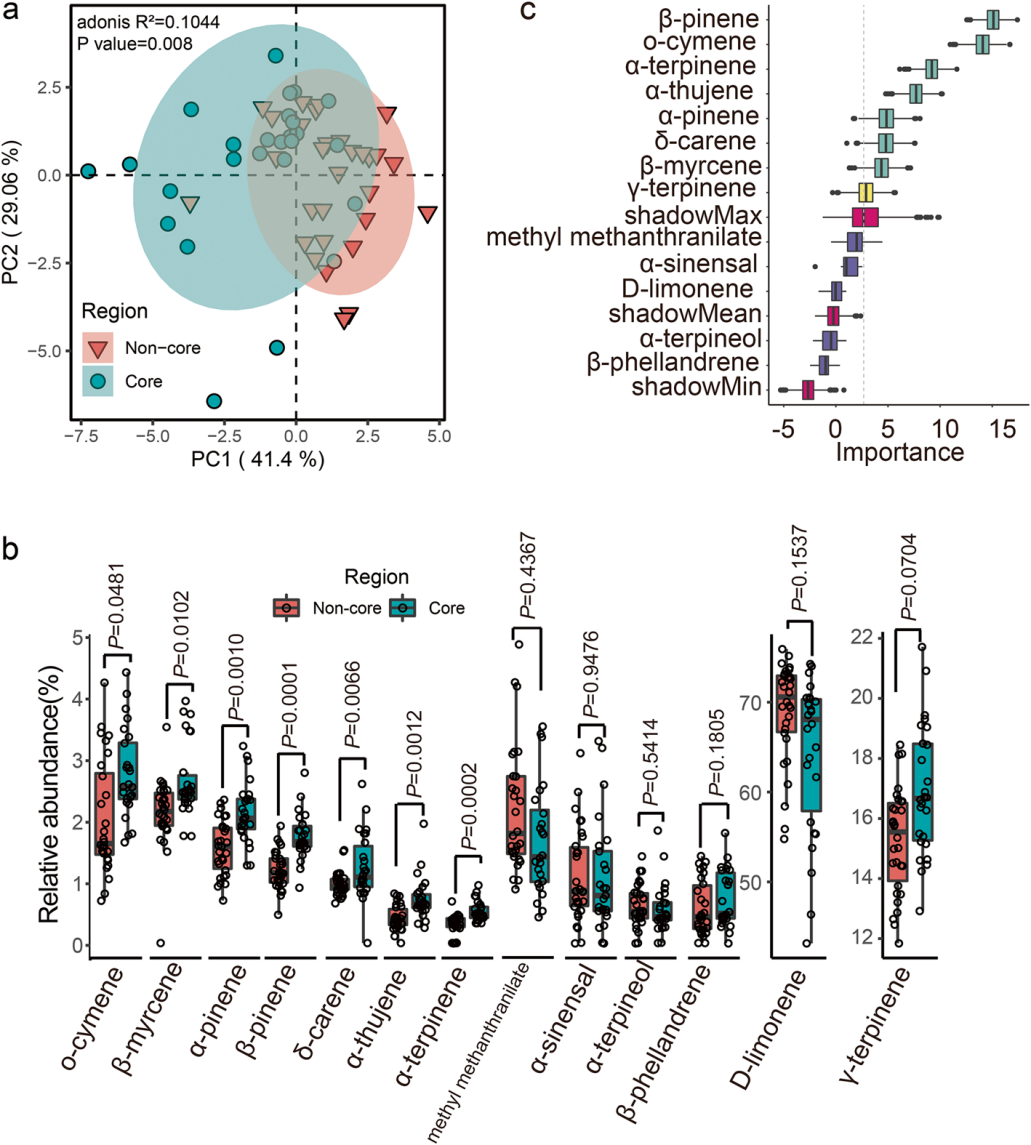
Comparative analysis of essential oils in the fruit peel of *C. reticulata* ‘Chachi’ in different planting areas. **a** Principal component analysis of Euclidean distance for essential oil contents in core and non-core region samples. **b** Boxplot showing the contents of essential oils between core and non-core regions. *n* = 56 biologically independent samples. Significance is indicated between core and non-core regions by the Wilcoxon rank sum test with an adjusted *P* value. **c** The importance rank of variables selected by the random forest model. Seven monoterpenes (green box) with high importance ranks were used to predict the source of *C. reticulata* ‘Chachi’

To explore the difference in essential oils between the seven representative orchards from the core and non-core regions (Table S1), gas chromatography–mass spectrometry (GC–MS) was used for qualitative analysis of the essential oils in the peel of *C. reticulata* ‘Chachi’. A total of 18 compounds were detected in *C. reticulata* ‘Chachi’ (Table S2). Principal component analysis (PCA) showed that the composition of essential oils in the core region was clearly separated from that in non-core regions (Fig. 1a). Among the 13 essential oils that were widespread in the citrus peel from core and non-core regions, seven were abundant in the core region, including o-cymene, β-myrcene, α-pinene, β-pinene, δ-carene, α-thujene and α-terpinene (Fig. 1b). It is worth noting that all seven essential oils are monoterpenes. Thus, to link these monoterpenes’ composition with citrus planting area, leave-one-out cross-validation (LOOCV) with a random forest algorithm was performed based on all 56 samples to select essential oils as features of citrus peel. We used 55 samples as a training set to perform feature selection and built a random forest classifier. Then, we used the classifier to predict the remaining sample and recorded the importance rank for each feature. Following the testing of each sample, seven essential oil components were designated as markers based on their importance rankings (Fig. 1c), which can predict the citrus planting area with an average accuracy of 82.86%. These results implied that citrus peel from the core region exhibited a special pattern of monoterpene components.

### Gene expression patterns of peels, leaves and roots

**Fig. 2:**
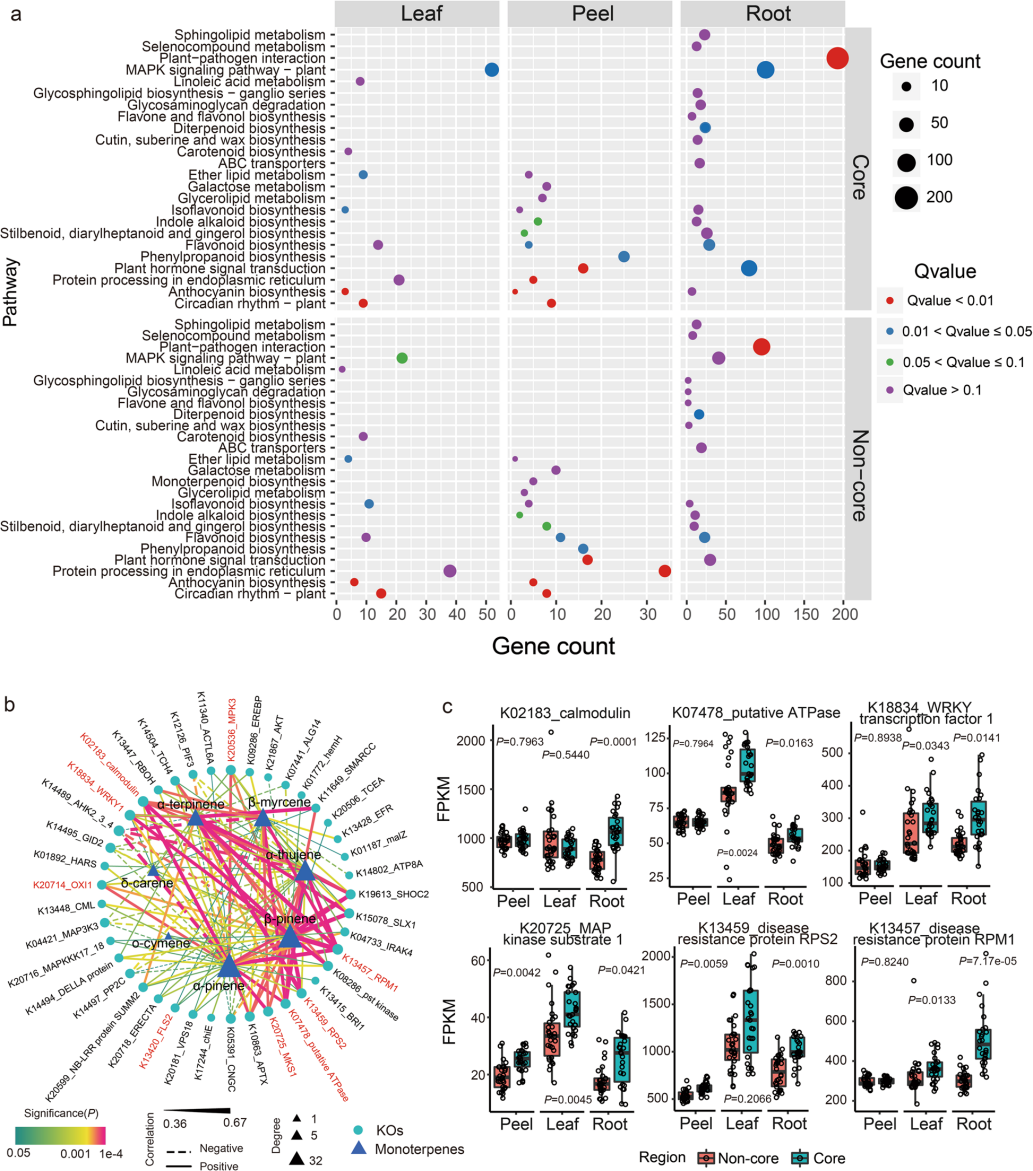
Highly expressed genes in the core region were correlated with the content of monoterpenes. **a** KEGG enrichment analysis of differentially expressed genes in peels, leaves and roots from the core region compared with the non-core region. The upper part of the figure displays the metabolic pathway enriched by the upregulated genes in core regions, and the lower part represents metabolic pathways enriched by the upregulated genes in non-core regions. Node size represents the number of upregulated genes within the pathway, and colour represents the significance of the pathway. ‘Gene count’ represents the number of genes enriched in a pathway. **b** Correlation-based network between root gene expression (nodes) and monoterpenes (triangles). Node size corresponds to the degree of each monoterpene. The thickness and colour of the edges denote the strength and significance, respectively. Solid and dashed edges indicate positive and negative correlations, respectively. **c** Cumulative relative abundance of six genes enriched in root samples. Significance is indicated between core and non-core regions by the Wilcoxon rank sum test

To preliminarily investigate the impact of planting area on the production of monoterpenes in citrus peel, we collected samples from leaves, peels and roots of *C. reticulata* ‘Chachi’, performed RNA-seq analysis and compared the transcriptome data between the core and non-core regions. A total of 1.1 terabases (Tb) of high-quality data from 167 samples (54 peels, 57 leaves and 56 roots) were obtained. A total of 21,645, 21,240 and 21,686 genes in leaves, peels and roots, respectively, were identified based on the gene set of the reference genome [35]. The differentially expressed genes (DEGs) in leaves, peels and roots between the core and non-core regions were identified. Kyoto Encyclopedia of Genes and Genomes (KEGG) pathway enrichment analysis revealed that the upregulated genes in the core region were mainly involved in plant and pathogen interaction, MAPK-signalling pathway, glycosaminoglycan degradation, phenylpropanoid biosynthesis and photoperiod response (circadian rhythm). The upregulated genes in the non-core region were mainly related to the functions of plant and pathogen interaction, ABC transporters, phenylalanine metabolism, secondary compound biosynthesis, protein metabolism and photoperiod response (circadian rhythm) (Fig. 2a). A greater number of genes related to biotic and abiotic stress responses were found in root samples from the core region than in those from the non-core region (Fig. 2a). These data indicated that core and non-core regions might differ in their biotic and abiotic environmental conditions.

To decipher the detailed information of highly expressed genes from the core region, 406, 1128 and 3212 genes with significantly higher transcripts were identified in the peels, leaves and roots, respectively (Fig. S1a). The genes of the monoterpene biosynthesis and terpenoid backbone biosynthesis pathways related to seven differentially enriched essential oil components were analysed in detail. Only the β-pinene synthase gene from the monoterpene biosynthesis pathway was found in peel samples, but there was no difference between core and non-core regions. Interestingly, the gene encoding 1-deoxy-D-xylulose-5-phosphate synthase (*DXS*), which is thought to catalyze the critical step in the terpene backbone biosynthesis pathway [36], was found to be highly expressed in leaves and peel in core region samples, and its expression level in leaves and root was higher than that in peels (Table S3, Fig. S1b). The expression of *DXS* was also verified by qRT–PCR, and the results showed that the expression pattern of *DXS* was similar to that of RNA-seq (Fig. S1c). These findings indicated high levels of transcripts related to monoterpene biosynthesis in core region samples.

To explore the relationship between highly expressed functional genes and seven monoterpene components abundant in the core region, a correlation-based network between functional KEGG orthologues (KOs) from leaves, peels and roots and essential oil components was constructed separately (Fig. 2b, Fig. S2). In the root network, the KOs of calmodulin showed a high correlation with six monoterpenes (Table S4), namely, β-pinene (*R*^2^ = 0.58), α-pinene (*R*^2^ = 0.58), α-thujene (*R*^2^ = 0.50), β-myrcene (*R*^2^ = 0.50), α-terpinene (*R*^2^ = 0.50) and δ-carene (*R*^2^ = 0.44). It has been proven that plant monoterpene production can be triggered by calmodulin signals, which play a pivotal role in the response to salt stress [37, 38]. In addition, the gene encoding putative ATPase, which was located on the plasma membrane and showed salt-tolerance activity, was also positively correlated with α-pinene, α-terpinene, β-myrcene, α-thujene and β-pinene. In addition, several pathogen resistance genes, such as *FLS2*, *WRKY1*, *MPK3*, *MKS1*, *OXI1*, *RPS2* and *RPM1*, were positively correlated with monoterpenes and overrepresented in root samples of the core region (Fig. 2c). Several sets of biotic and abiotic stress-responsive genes were highly associated with the monoterpene content. Similar patterns were also observed in leaves and peels, for example, the disease resistance genes *RPS2*, *PR1*, *WRKY29*, *SUMM2*, *MKS1* and *VSP2*. Among these, *WRKY29*, which is involved in the expression of defence genes in the innate immune response of plants, could bind to the promoter region and regulate the expression of *DXS*, according to transcription factor prediction using JASPAR. In particular, the abscisic acid receptor gene *PYR/PYL*, which responds to salt and drought stress, was found to be significantly positively correlated with the contents of α-pinene, α-thujene, β-myrcene and α-terpinene in leaf samples. Typically, these data illustrated that the genes induced by biotic and abiotic stress correlate with the production of monoterpenes in core regions, suggesting that unique soil properties and microbial environmental conditions might affect the accumulation of monoterpenes.

### The special features of soil properties in the core region

**Fig. 3:**
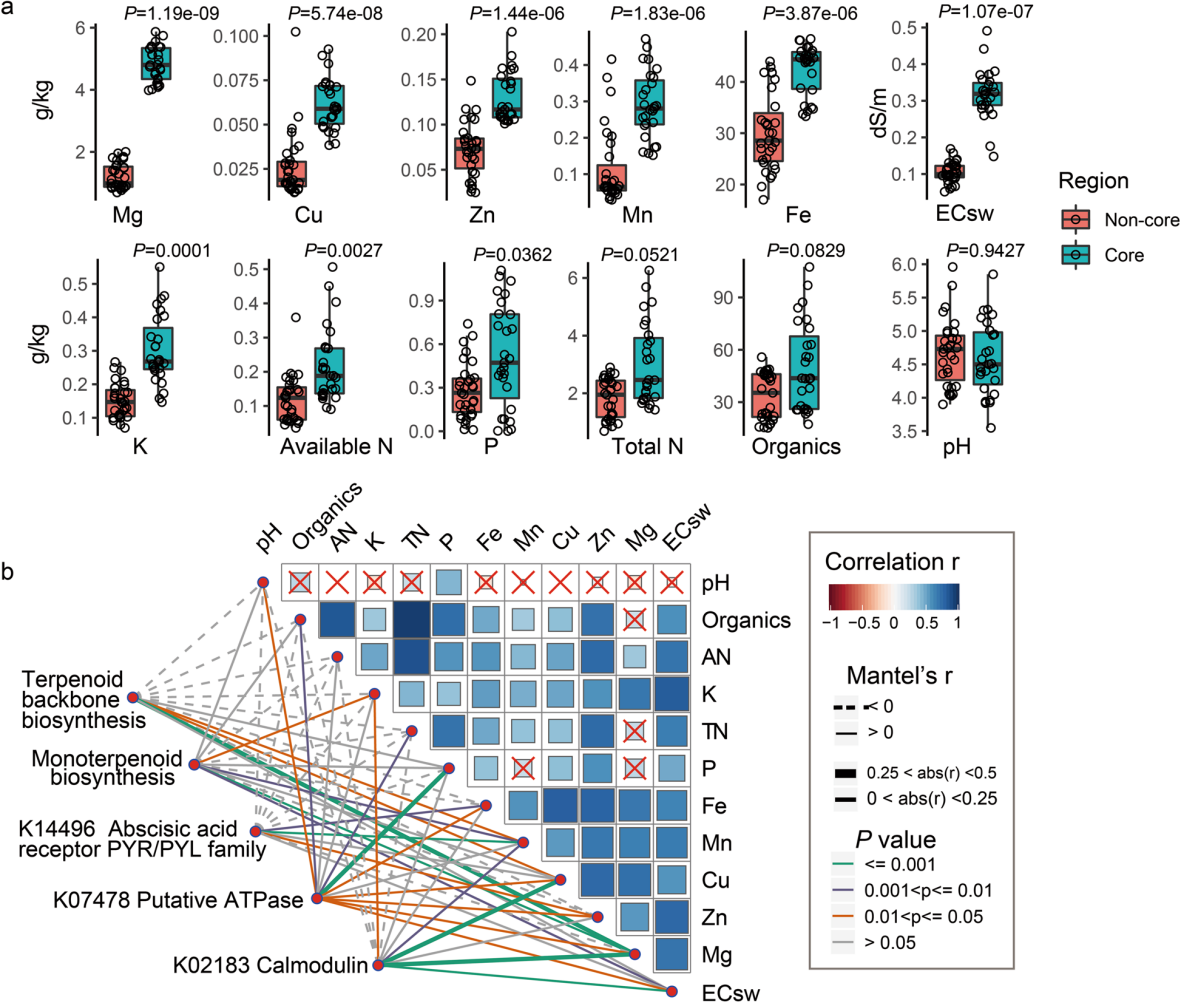
Soil chemical properties and their correlations with root gene expression. **a** Soil physical and chemical properties in rhizosphere soil samples from the core region differed from those in samples from the non-core region. Significance is indicated between core and non-core regions by the Wilcoxon rank sum test. AN, available nitrogen; TN, total nitrogen; EC_sw_, electrical conductivity of soil water. **b** Pairwise comparisons of environmental factors are shown, with a colour gradient denoting Spearman’s correlation coefficient. Root transcripts were related to each environmental factor by partial (geographic distance-corrected) Mantel tests. Edge width corresponds to Mantel’s R statistic for the corresponding distance correlations, and edge colour denotes the statistical significance based on 9999 permutations. Solid and dashed edges indicate positive and negative correlations, respectively

Soil nutrients are important for plant growth and development. To compare the soil conditions between core and non-core regions, 12 soil physical and chemical properties were detected, including the macro-element nutrients (N, P, K and Mg), micro-element nutrients (Fe, Mn, Zn and Cu), organics, pH and electrical conductivity of soil water (EC_sw_) (Table S5). Comparative analysis showed that soil nutrients in core regions differed from those in non-core regions, illustrated by the higher content of available nitrogen (AN), total nitrogen (TN), P, K, Mg and micro-element nutrients (Fig. 3a). Specifically, the core region shows an average of 3.99 × , 2.56 × , 2.35 × and 1.40 × higher Mg, Mn, Cu and Fe levels than the non-core region, respectively. Moreover, the EC_sw_ in the core region was 3.02 × higher than that in the non-core region. These data illustrated that the soil of the core region is rich in both macro- and micro-elements (Mg, Mn, Cu, Fe) coupled with higher soil salinity.

Mantel’s test was used to investigate the relationship between soil properties and the expression of genes related to monoterpene biosynthesis and salt stress responses in roots. None of the macro-element nutrients (N and P), pH, or organic factors were found to be associated with terpenoid backbone biosynthesis, whereas only K showed a positive correlation with monoterpene biosynthesis (Fig. 3b). However, the micro-element Mn, macro-element Mg and EC_sw_ contents showed a significant positive correlation with terpenoid backbone biosynthesis and the monoterpene biosynthesis pathway. Mn and Mg are important cofactors for enhancing the activities of β-pinene synthase and *DXS* [39]. These correlations suggested that these environmental parameters of the core region could improve the production of monoterpene, especially the micro-element Mn, the macro-element Mg and EC_sw_. As described above, the salt tolerance gene calmodulin and responsive gene abscisic acid receptor *PYR*/*PYL* family were highly interrelated with the content of monoterpenes and were also found to be significantly correlated with the contents of K, Mn, Mg, Cu and P; EC_sw_; and pH. Collectively, the special soil conditions in the core region, such as higher micro-elements (Mg, Mn, Cu and Fe), higher macro-elements (K and P) and higher salinity, enhanced monoterpene biosynthesis gene and abiotic tolerance gene expression, directly or indirectly contributing to the production of monoterpenes.

### Microbial taxonomic and functional features

Rhizosphere and associated bulk soil samples of *C. reticulata* ‘Chachi’ were collected from seven representative orchards in core and non-core regions to explore the identity of the microbes constituting the rhizosphere microbiome, as well as their genomic and, consequently, functional features. De novo assembly was performed using a groupwise approach. Bacteria showed the highest abundance in all rhizosphere samples, with a total of 159 bacterial phyla (Table S6) and 2332 bacterial genera (Table S7). Among all the bacterial phyla, Proteobacteria accounted for 14.45 to 65.41% of the total abundance in each sample, followed by Actinobacteria (11.32 to 59.97%) and Acidobacteria (3.82 to 40.45%) (Fig. S3).

**Fig. 4:**
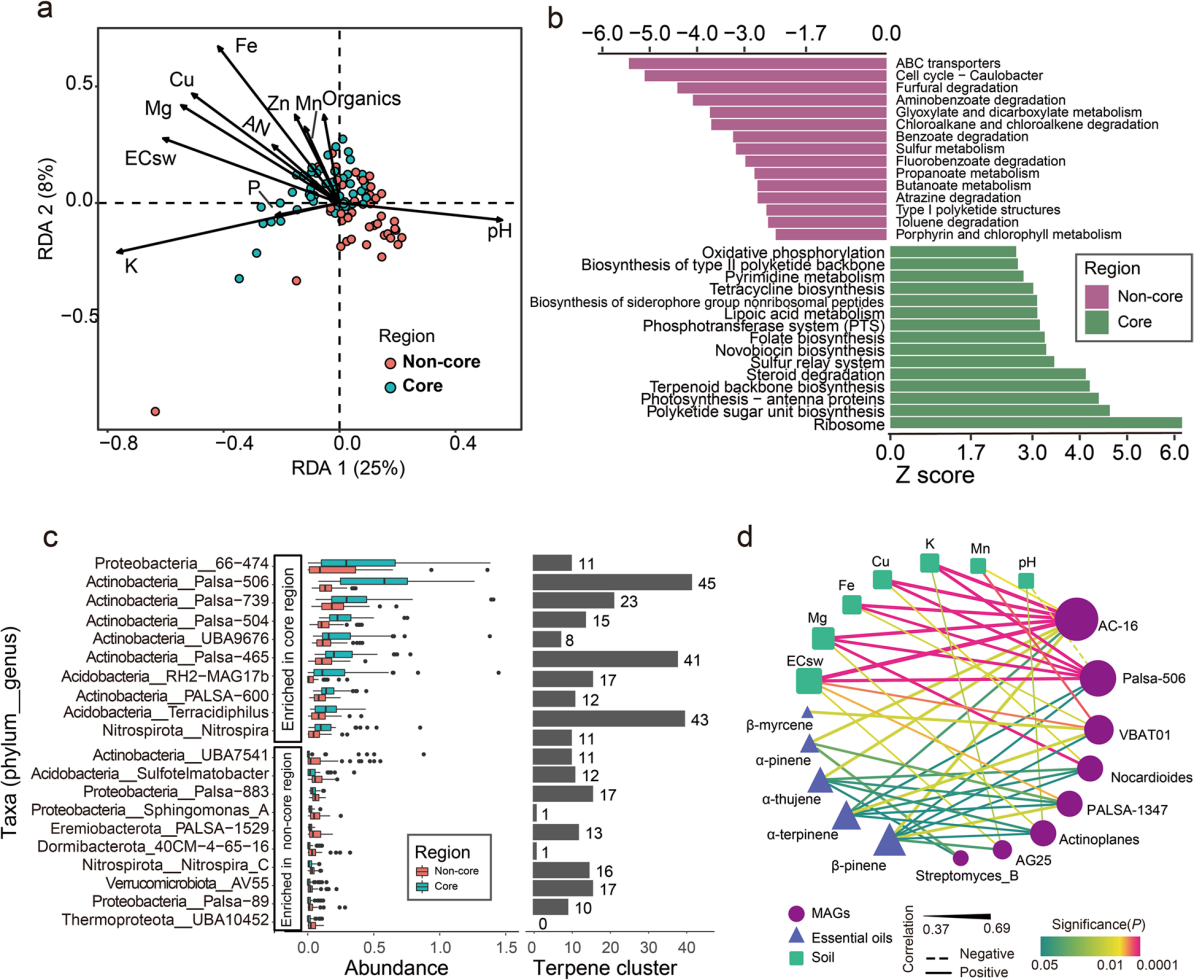
Functional characteristics of differential bacteria between core and non-core rhizosphere soil microbiotas. **a** Environmental factors (K, ECsw, Mg, Cu, Fe, Zn, Mn, P, AN, organics and pH) significantly explained the observed compositional variation in the microbial community (genus level) in rhizosphere soil samples. **b** KEGG pathways compared between core and non-core regions. Pathways with a significant difference in |*z* score|≥ 1.7 are shown. **c** Differential enrichment of MAG genera between regions was identified using a Wilcoxon rank sum test with an adjusted *P* value of 0.05. Only the top 10 enriched MAG genera are shown in the figure. The terpene cluster number in each genus is shown on the right. **d** A network was constructed by correlation of MAGs (purple nodes), monoterpenes (blue triangles) and soil properties (green square). Node size corresponds to the degree of each node. The thickness and colour of the edges denote strength and significance, respectively. Solid and dashed edges indicate positive and negative correlations, respectively

Unconstrained principal coordinate analysis (PCoA) was performed to uncover the separate patterns between microbial communities based on the Bray–Curtis distance of taxonomic genus abundance (Fig. S4a). The majority of variations in microbial composition were explained by geographical locations. Permutational multivariate analysis of variance (PERMANOVA) corroborated that geographical location had the largest impact on microbial composition, explaining 11.83% of the variation within the microbiome data, followed by microhabitat (the habitat of the microorganism derived from bulk soil or rhizosphere), which explained 6.75% of the variation. RDA was performed to discern the relationship between the microbial community structure and soil properties. The compositional variations in the core region microbiome were significantly explained by soil factors (Fig. 4a), such as pH, organics, available nitrogen (AN), K, P, Fe, Mn, Cu, Zn, Mg and EC_sw_. In particular, K, EC_sw_, Mg, Cu and Fe exhibited a strong positive correlation with most samples from the core region, while pH displayed a strong positive correlation with most samples from non-core regions. Mantel’s test also verified that these soil factors were correlated with microbial taxonomic and functional compositions (Fig. S4b). Collectively, soil factors such as K, EC_sw_, Mg, Cu and Fe in the core region not only were associated with the gene expression of citrus but also impacted the microbial composition of rhizosphere soil.

The differentially abundant microbial genera and KOs were revealed by pairwise comparison of rhizosphere samples of core regions and non-core regions separately. A total of 389 genera were abundant in core regions, while 244 genera were enriched in non-core regions. Of these, Actinobacteria and Chloroflexi had greater numbers of genera enriched in the core region (Fisher test, *P* value < 0.05). The differential KOs mainly contributed to 50 pathways. Of these pathways, 26 were enriched in rhizosphere samples of the core region, while 24 were enriched in the other region (Fig. 4b). Of these functional traits, ribosome, polyketide sugar unit biosynthesis, photosynthesis-antenna proteins, terpenoid backbone biosynthesis and steroid degradation were significantly elevated in the rhizosphere microbiome of the core region. The terpenoid backbone biosynthesis pathway was enriched in the rhizosphere microbiome of the core region compared to the non-core regions, indicating that the genes for terpenoid backbone biosynthesis in soil bacteria from the core region were more abundant than those in the non-core region, which might provide intermediates to promote the production of monoterpenes in plants. F-type ATPase was reported to catalyze the hydrolysis or synthesis of ATP coupled with H^+^ (or Na^+^) transport across a membrane and to respond under high salinity [40, 41]. Four genes encoding F-type ATPase were enriched in core rhizosphere soil samples (Fig. S4c), suggesting that the rhizosphere soil microorganisms in the core region possessed a high level of salt tolerance to adapt to the high-salinity soil in this region.

Second, to explore the taxonomic and functional compositions of the rhizosphere soil microbiome at the genome level, we reconstructed 28,570 metagenome-assembled genomes (MAGs) from the 92 metagenomes. This set was narrowed to 6075 MAGs after consideration of completeness and contamination. After dereplication, a final set of 3350 MAGs was generated and used in subsequent analyses, recovering 23 phyla and 352 genera (Table S8). Comparative analysis was performed to identify the differentially enriched MAG genera between the core region and non-core regions. Seventy-five bacterial genera, such as *66–474* (Proteobacteria), *Palsa-506* (Actinobacteria), *Palsa-739* (Actinobacteria), *Palsa-504* (Actinobacteria) and *UBA9676* (Actinobacteria), were observed with higher abundance in the core region, while 79 bacterial genera, such as *UBA7541* (Actinobacteria), *Sulfotelmatobacter* (Acidobacteria), *Palsa-883* (Proteobacteria), *Sphingomonas* (Proteobacteria) and *PALSA-1529* (Eremiobacteria), were abundant in the non-core region (Fig. 4c, Table S9). Twenty-five of 75 enriched genera in the core region were from Actinobacteria, suggesting that Actinobacteria had a greater number of MAG genera enriched in core regions than in non-core regions.

Subsequently, the relationships between soil chemical properties, differentially enriched MAG genera and the content of monoterpenes were explored using Spearman’s rank test (Fig. 4d). Eight MAG genera enriched in the rhizosphere soil of the core region showed significant positive correlations with five monoterpenes (α-pinene, α-thujene, β-pinene, α-terpinene and β-myrcene). Among these, five genera from Actinobacteria, *AC-16*, *Actinoplanes*, *Nocardioides*, *Palsa-506* and *Streptomyces*, were positively correlated with four monoterpenes, α-pinene, α-thujene, β-pinene and α-terpinene. In addition, *VBAT01* from Bacteroidetes was positively correlated with β-pinene, β-myrcene and α-terpinene; *PALSA-1347* from Planctomycetota was positively correlated with α-pinene, α-thujene, β-pinene and α-terpinene; and *AG25* from Gemmatimonadota was positively correlated with β-pinene. Moreover, soil chemical properties such as EC_sw_, Mg, Cu, Fe, K and Mn showed a significant positive correlation with these microbial genera. A total of 169, 85 and 42 genes that function as F-type ATPases were found in the genera *Streptomyces*, *Nocardioides* and *Actinoplanes*, respectively, implying that these microbes are tolerant to salt in high-salinity environments. These results illustrated that soil condition-determined rhizosphere microbes might contribute to the accumulation of monoterpenes in citrus peels. Furthermore, we found that plant immunity genes (including *FLS2*, *SUMM2*, *RPS2*, *OXI1* and *RPM1*) correlated with the content of monoterpenes were also significantly correlated with eight genera that were enriched in rhizosphere soils (Fig. S5).

### Synthetic communities boost the accumulation of monoterpenes in the leaves of *C*. *reticulata* ‘Chachi’ in an immune-dependent manner

**Fig. 5:**
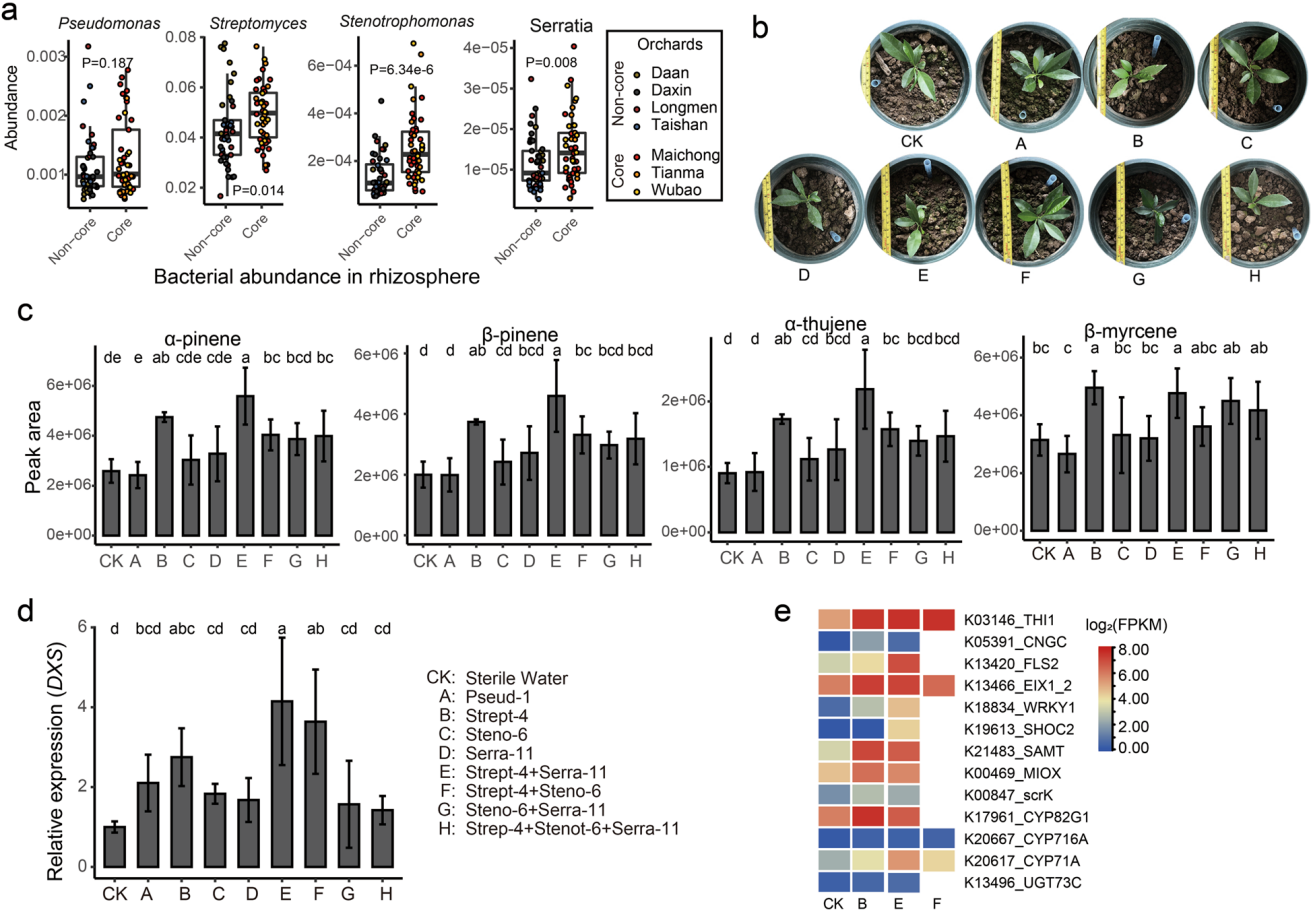
Synthetic communities boost the accumulation of monoterpenes in the leaves of *C. reticulata* ‘Chachi’. **a** Relative abundance of *Pseudomonas*, *Streptomyces*, *Stenotrophomonas* and *Serratia* in the rhizosphere. Significance is indicated between core and non-core regions by the Wilcoxon rank sum test. **b** The treatment of nine groups of *C. reticulata* ‘Chachi’ with different treatments. Strep-4 from *Streptomyces*, Steno-6 from *Stenotrophomonas*, Pseud-1 from *Pseudomonas* and Serra-11 from *Serratia*. **c** The content of four monoterpenes in *C. reticulata* ‘Chachi’ leaves after inoculation with Strep-4 and its SynComs. Different letters denote a significant difference (LSD, *P* < 0.05). **d** The relative abundance of the *DXS* gene in *C. reticulata* ‘Chachi’ leaves was measured by qRT–PCR. **e** Differentially expressed genes in citrus leaves after inoculation with Strep-4 and its SynComs

To verify the effects of bacteria enriched in core region soil on the production of monoterpenes in citrus, a range of taxonomically different bacterial strains were isolated from the rhizosphere soil and roots of *C. reticulata* ‘Chachi’ in the core region. The bacterial isolates were sequenced by a multi-barcode method to target the V4 region of the 16S rRNA gene on the BGISEQ platform. A total of 94 and 215 bacteria from rhizosphere soil and roots were retained, respectively, representing four bacterial phyla (Actinobacteria, Proteobacteria, Bacteroidetes and Firmicutes) and 16 genera (Table S10). Of these, the genera *Streptomyces*, *Stenotrophomonas* and *Serrata* showed higher abundance in the soil of the core region, with *Pseudomonas* and *Streptomyces* from the actinobacteria phylum also showing a positive correlation with the content of monoterpenes. To further verify the effect of bacteria enriched in the core region on the synthesis of monoterpenes in *C. reticulata* ‘Chachi’, we designed SynCom experiments based on three soil-derived strains (Strep-4 from *Streptomyces*, Steno-6 from *Stenotrophomonas* and Pseud-1 from *Pseudomonas*) and one endophyte strain, Serra-11, from *Serrata* (Fig. 5a). After inoculating the bacterial strains and their SynCom into *C. reticulata* ‘Chachi’ planted in sterilized soil for 30 days (Fig. 5b), the contents of essential oil in citrus leaves were detected using the GC–MS method. Six differential monoterpenes were observed, and the contents of four monoterpenes, α-pinene, β-pinene, α-thujene and β-myrcene, significantly increased in the citrus leaves inoculated with *Streptomyces* (Group B) and its SynCom (Groups E and F) compared with the non-inoculated samples (Fig. 5c). However, the contents of the monoterpenes showed no difference between the treatments with the other three bacterial strains (Steno-6, Serra-11 and Pseud-1) alone and the control. The highest contents of α-pinene, β-pinene and α-thujene were detected in the treatment with the combination of Strep-4 and Serra-11 (Group E). These results indicated that *Streptomyces* Strep-4 plays a major role in promoting monoterpene production in citrus leaves and that *Serrata* Serra-11 could strengthen the effects of *Streptomyces* Strep-4 on monoterpene accumulation in citrus. The expression abundance of the *DXS* gene was also quantified using qRT–PCR, and it was found to be significantly enhanced upon treatment with *Streptomyces* and its SynCom (Fig. 5d). These results verified that bacterial strains from the core region could boost the accumulation of monoterpenes in *C. reticulata* ‘Chachi’.

To shed light on how bacterial strains affect monoterpene accumulation in citrus, we analysed the transcriptomes of citrus leaves colonized for 30 days by *Streptomyces* Strep-4 (Group B) and its SynCom (Groups E and F), which show a good promotion effect on monoterpene production. The differentially expressed genes in strain-inoculated versus non-inoculated samples were identified. A total of 129, 78 and 330 genes were highly expressed in response to treatment with Strep-4 (B) and its SynCom (E and F), respectively. We aggregated these genes on the basis of their KO functions. Consistent with the observations, a subset of genes related to terpenoid biosynthesis and modifications (*CYP82G1*, *CYP716A*, *CYP71A*, *UGT73C* and so on) were highly expressed in the treatments (Fig. 5e, Table S11). Intriguingly, a major group of functional genes involved in the plant immune response, such as *SAMT*, *CNGC*, *FLS2*, *EIX1_2*, *SHOC2* and *WRKY1*, were also highly expressed. Among these, SAMT (salicylate 1-O-methyltransferase) showed higher abundance in the treatments, which could biologically convert salicylic acid (SA) to methyl salicylate (MeSA). *MeSA* is a critical signal gene that can induce systemic acquired resistance (SAR) in plants [42]. These results illustrated that *Streptomyces* Strep-4 in the core region and its SynCom might activate terpene synthesis and transformation and promote monoterpene accumulation in *C. reticulata* ‘Chachi’ in an immune-dependent manner.

### The potential for secondary metabolite biosynthesis of microbes in rhizosphere soil

To explore the potential for secondary metabolites in rhizosphere soil microorganisms, 10,672 biosynthetic gene clusters (BGCs) were identified for contigs of > 10 kb within the set of 3350 dereplicated MAGs. The most common types of BGCs identified in our dataset were terpenes (2317 BGCs), NRPS (2062 BGCs), NRPS-like (1159 BGCs), bacteriocins (1084 BGCs) and T1PKS (1076 BGCs) (Fig. S6). A large fraction (64.80%) of terpene gene clusters originated from Actinobacteria (23.36%), Proteobacteria (20.81%) and Acidobacteria (20.63%) (Table S9). Interestingly, a total of 391 terpene BGCs were observed in 47 bacterial genera enriched in the core region, while 227 terpene BGCs from 48 genera were enriched in the other region (Wilcox rank test, adjusted *P* < 0.05). Moreover, the abundance of terpene BGCs was significantly higher in the core region than in the non-core region (Fig. S7, Wilcox rank test, *P* = 9e − 4). Of the 391 terpene BGCs from core region-enriched genera, 219 were from Actinobacteria genera. In particular, the genus *Palsa-506* (Actinobacteria), with 45 terpene BGCs, was abundant in the core region (Fig. 4c). These results indicated that more terpene BGCs were found in bacterial genera enriched in rhizosphere soil from the core region, especially the genera from Actinobacteria.

To further understand the genomic potential for terpene biosynthesis in rhizosphere soil microbes, three soil-derived bacterial strains used in the above experiments were sequenced and assembled. The genome sizes of Pseud-1, Strept-4 and Steno-6 were 6.1 Mb, 8.4 Mb and 8.5 Mb with scaffold N50 values of 263 Kb, 449 Kb and 316 Kb, respectively. Interestingly, 4 terpene clusters were found in Strept-4, and 1 in Pseud-1 and Steno-6 (Table S12). A complete terpenoid backbone pathway was found in all three strains, including *DXS* genes, indicating that the strains have the ability to biosynthesize the precursor of monoterpenes. As soil microbes could enter the root, we hypothesized that these microbes might directly colonize the roots of citrus to synthesize the intermediate product of monoterpenes.

### Root endophytes and their potential to produce bioactive compounds

**Fig. 6:**
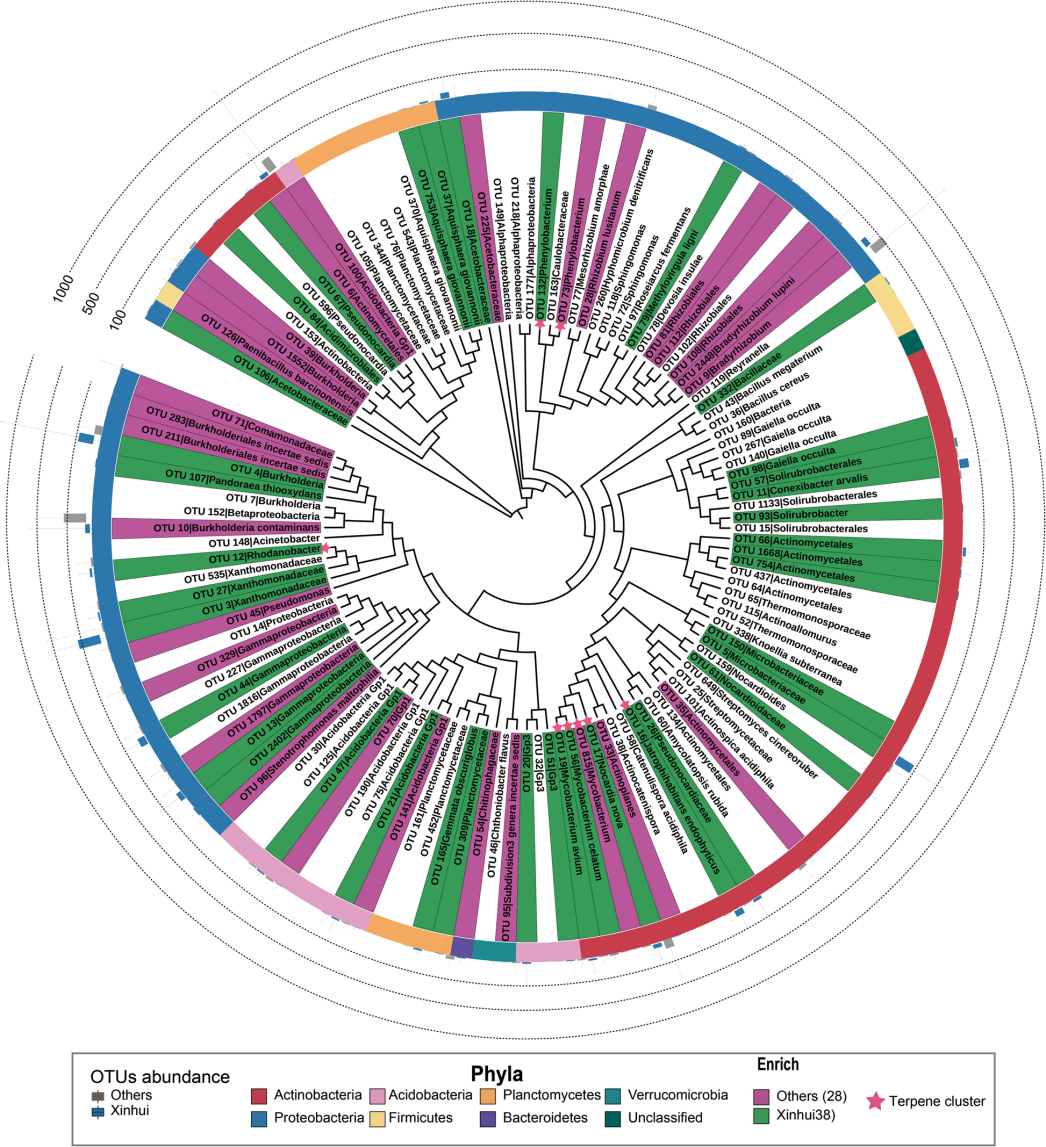
Composition of the endophyte microbiome. Phylogenetic tree of the 122 core OTUs. Stars represent the OTUs containing a terpene cluster. The colour of leaf labels represents OTUs enriched in the core region (green) and non-core region (purple). The inner ring represents the microbial OTU phylum information. The box in the outer ring represents the abundance of each OTU, the blue boxes represent the core region, and the grey boxes represent the non-core region

To validate our hypothesis, we performed 16S rRNA gene amplicon sequencing to unravel the composition of bacterial endophytes in root samples, and 19,976,393 high-quality sequences were generated and clustered into 2570 operational taxonomic units (OTUs). PCoA based on OTU composition revealed that the endophyte microbiota of core and non-core regions formed two distinct clusters, which were separated along the first coordinate axis (Fig. S8), suggesting the distinct endophytic composition in the root sample of the core region. Fifty-two endophyte OTUs were annotated to the same genera that were enriched in the rhizosphere soil of the core region, specifically the genera *Streptomyces*, *Mycobacterium*, *Rhodanobacter*, *Amycolatopsis*, *Jatrophihabitans* and so on (Fig. S9), indicating the possibility that endophyte microbes enter the root from rhizosphere soil. A total of 122 endophyte OTUs were identified according to an 80% occurrence frequency in all samples, mainly comprising seven phyla, such as Proteobacteria and Actinobacteria. Of these, 38 and 28 microbial OTUs were enriched in the core region and non-core region, respectively (Fig. 6, Table S13). Twenty-one core region-enriched endophyte OTUs were positively correlated with 4 of 7 differentially enriched monoterpenes, such as β-pinene, α-terpinene, o-cymene and δ-carene (Table S14). Specifically, OTU_37 (Aquisphaera) was correlated with four monoterpenes. In addition, OTU_4 (Burkholderia), OTU_332 (Bacillaceae), OTU_165 (Gemmata), OTU_309 (Planctomycetaceae), OTU_21 (Gp1), OTU_16 (*Jatrophihabitans*) and OTU_56 (*Mycobacterium*) were positively correlated with three monoterpenes (β-pinene, α-terpinene and δ-carene). Similarly, OTU_19 (*Mycobacterium*) and OTU_79 (*Methylovirgula*) were positively correlated with β-pinene and δ-carene, respectively. Interestingly, among the seven differential monoterpenes, three monoterpenes were correlated only with rhizosphere soil microbes, while two monoterpenes were correlated only with endophytic bacteria and two were correlated with both. This implies that rhizosphere soil microbes and endophyte microbes exert different functions in the accumulation of monoterpenes in *C. reticulata* ‘Chachi’.

To confirm that root endophytes are derived from soil microorganisms, we first matched the MAG-derived 16S rRNA sequences to the 16S rRNA amplicon dataset from the root endophyte microbiota. As a result, 39 MAGs contained complete 16S rRNA genes, 14 of which matched 13 endophyte OTUs (Table 1). Of these, the MAG UBA5177 from the Chloroflexota phylum with 100% 16S rRNA similarity with the endophyte OTU_674 showed higher abundance in the rhizosphere soil of the core region and contained 2 terpene BGCs. These results implied that some microbes enter the root from rhizosphere soil and facilitate the synthesis of terpenes in citrus.

**Table 1:** MAG 16S rRNA gene and OTU sequence alignment results

Number	OTU id	MAG id	Identity (%)
1	OTU_226	group.F.bin.1163	98.7
2	OTU_411	group.C1.bin.187	97.2
3	OTU_431	group.C1.bin.50	100
4	OTU_552	group.B.bin.1488	98.4
5	OTU_552	bins.group.C2.079	98.4
6	OTU_619	group.B.bin.345	100
7	OTU_674	group.G.bin.1731	100
8	OTU_863	group.D.bin.868	97.2
9	OTU_1006	bins.group.G.1275	100
10	OTU_1261	bins.group.C1.065	99.6
11	OTU_1624	group.I1-A.bin.349	97.2
12	OTU_2028	bins.group.D.0077	97.8
13	OTU_2117	group.D.bin.868	97.6
14	OTU_2490	group.B.bin.300	100

Second, we mapped the endophyte OTUs to the 16S rRNA V4 regions of root-derived strains and soil-derived strains from the core region. A cultivated strain was considered a representative OTU if its 16S rRNA gene had 97% similarity with the endophyte OTU, resulting in 35 endophyte OTUs with representative strains (Table S15). Of these, 23 endophyte OTUs with representative strains were isolated from the citrus root and 12 endophyte OTUs with representative strains were derived from the rhizosphere soil. These results further validated that some endophyte microbes entered the root from the rhizosphere soil. Among these, two OTUs (Planococcaceae OTU 332 and Burkholderia OTU 4) with representative strains were positively correlated with monoterpenes, indicating that these strains might function in monoterpene production.

To investigate the functions of endophyte microbes of *C. reticulata* ‘Chachi’ in the core region, an endophyte strain, *Serratia marcescens* strain Serra-11 (OTU_196), was sequenced and de novo assembled with a genome size of 5.1 Mb (Fig. S10a), comprising 4875 functional genes. A complete terpenoid backbone pathway was annotated, indicating that the strain has the ability to biosynthesize the precursor of monoterpenes in roots (Fig. S10b). This observation further verified that the endophyte bacteria could promote the monoterpene content by synthesizing the intermediate product.

To explore the expression pattern of the microbial BGCs in different tissues of *C. reticulata* ‘Chachi’, the RNA sequences from the roots, leaves and peels of *C. reticulata* ‘Chachi’ were mapped to the BGC dataset predicted from the metagenome data, and the RPKMs of the mapped BGCs were calculated using BiG-MAP [43]. A total of 47 BGCs were mapped and expressed in citrus peel, leaf and root, mainly including terpenes (11) and NRPS (10). In particular, 9 of 11 microbial terpene clusters were highly expressed in root samples, only 1 terpene cluster was highly expressed in leaf samples, and 1 terpene cluster was highly expressed in peel samples (Fig. S11), which might be due to the higher microbial diversity in roots than in leaves and peels. Among the 9 terpene BGCs identified in roots, 7 came from Actinobacteriota and 2 from Proteobacteria. These results indicated that endophyte microorganisms in roots could synthesize terpenes, especially endophyte bacteria from Actinobacteriota.

To further investigate the potential for producing monoterpene from root endophyte bacteria, we performed Gram staining to observe the microstructure of root endophytic bacteria in *C. reticulata* ‘Chachi’ from the core region. Gram-positive bacteria were distributed in the cytoplasm and intercellular space of root cortical cells, while gram-negative bacteria were only distributed in the cytoplasm of root cortical cells (Fig. S12a-f). Based on transmission electron microscopy (TEM) observations, endophytic bacteria with different morphologies were also found to occur in the intercellular space and cytoplasm of root cortical cells and vascular cylinder cells; in particular, the presence of lipid droplets was observed in some bacterial cells (Fig. S12g-l). No endophyte bacteria were observed in the intercellular space and cytoplasm of the root cortical cells and vascular cylinder cells of aseptic seed culture plants (Fig. S12m-n). Moreover, lipid droplets were also found in the vascular cylinder and cortical cells of young and more mature roots, as well as in the vascular bundle in peels of *C. reticulata* ‘Chachi’ in the core region. However, few droplets were found in the vascular cylinder and cortical cells of aseptic seed culture plants (Fig. S13), once again indicating the capability of endophyte microorganisms to synthesize monoterpene.

## Discussion

In the present work, we linked soil properties, soil microorganisms and root endophytes to the production of essential oil by *C. reticulata* ‘Chachi’ from different geographical locations. We verified that the *Streptomyces* strain Strep-4 plays a major role in promoting monoterpene production in citrus leaves and that *Serrata* Serra-11 could strengthen the effects of *Streptomyces* on the monoterpene accumulation of citrus in the core region. These results provide a fundamental basis to explore the impact of soil conditions and the microbiome on the bioactive compounds of citrus peel.

Essential oils are the most important bioactive components of GCRP, with a relatively high abundance [44]. However, their compositions and differences in the peel of *C. reticulata* ‘Chachi’ between the core region and non-core region are not known. In this study, we identified seven monoterpenes abundant in the core region that differed from those in non-core regions. Thus, we focused on the causes of the accumulation of these seven monoterpenes in the citrus peel of the core region from three items: the host transcripts, soil chemical properties and root-associated microbiome. Due to the limitation of reference proteins of synthases of 7 monoterpenes, only β-pinene synthase was found in our transcriptomic data, but it showed no difference between the core and non-core regions. Interestingly, the *DXS* genes encoding key enzymes for the synthesis of precursors of monoterpenes from the terpene backbone biosynthesis pathway were highly expressed in leaves and roots of the core region, which might promote monoterpene production in the peel in the core region by providing numerous precursor substances. It has been reported that *DXS* strongly accumulates under salt stress in plants [45]. Consistently, there was a higher ECsw in the core region soil, which represented the higher salinity of the soil.

Plants employ terpenoid metabolites for several basic functions influencing growth and development but use the majority of terpenoids for more specialized chemical interactions and protection in response to their biotic and abiotic environments [46]. Consistently, differentially enriched genes between locations were mainly related to functions such as circadian rhythm, secondary metabolite biosynthesis, biotic and abiotic resistance and plant hormone signal transduction. Circadian rhythm has positive effects on regulating both structural genes and transcription factors for monoterpene biosynthesis [47]. Moreover, highly expressed genes such as calmodulin, putative ATPase, *MAPK1* and the abscisic acid receptor *PYR/PYL* family related to salt stress resistance in root and leaf samples from the core region were significantly correlated with the content of monoterpenes. These results indicate that high salinity of soil induces higher expression of abiotic resistance genes and may directly or indirectly promote the accumulation of monoterpenes in citrus peel. In fact, prolonged exposure to salt stress affects the accumulation of monoterpenes in *Artemisia annua* L. [48]. By analysing soil chemical properties, we also confirmed that the soil in the core region had a higher EC_sw_, as well as higher K, Mg and micro-element contents, than the soil in the non-core region. Of these, Mn and Mg as cofactors have been used to improve the synthase activity of monoterpenes [39]. Mantel’s test also demonstrated that Mg, Mn and EC_sw_ were positively correlated with monoterpenes as well as terpene backbone biosynthesis pathways. Based on these observations, we speculate that the high content of micro-elements, Mg and EC_sw_ in the core region contribute to the biosynthesis of monoterpenes in citrus peel.

In addition, a greater number of plant immune-responsive genes were induced in root, leaf and peel samples of the core region, such as *WRKY1*, *WRKY29*, *MKS1*, *MPK3*, *RPS2*, *RPM1*, *SUMM2* and *VSP2*, which were also positively correlated with the content of seven monoterpenes. A previous study also illustrated that *WRKY1* can regulate the sesquiterpene, diterpene and triterpene synthase genes by controlling critical rate-limiting steps [49]. *VSP2* is also a marker gene commonly used for JA induction, and the emission of several monoterpenes was enhanced in all JA-treated plants [50]. It would be interesting to study whether and how microorganisms regulate the production of monoterpenes through interactions with the plant immune system.

Therefore, we analysed taxonomic and functional structure and their association with the content of monoterpenes by exploring the rhizosphere soil and endophyte microbiome. We found that the soil properties were associated with the variations in rhizosphere soil microbiomes between the core and non-core regions, especially the micro-elements, Mg and EC_sw_. The terpene backbone biosynthesis pathway and salt tolerance genes were significantly enriched in the rhizosphere soil samples of core regions, indicating that the microorganisms in rhizosphere soils were rich in salt resistance and the capability to synthesize terpene. Eight MAG genera enriched in the core region rhizosphere soil showed positive correlations with soil chemical properties (such as EC_sw_, Mg, Cu, Fe, K and Mn), monoterpenes and plant immune genes. Several salt tolerance genes were found in three genera (*Streptomyces*, *Nocardioides* and *Actinoplanes)*. The findings suggested that these salt-resistant genera might promote monoterpene accumulation by triggering the plant defence response. Unlike the non-core region, the core region is located at the intersection of the sea and three rivers, where seawater flows back to the planting field every year. The special soil environment in the core region nourishes special microbial groups, which might be recruited by plants to the rhizosphere to promote the production of plant terpenoids. Root exudates and phytohormones play an important role in modulating microbial colonization of the root zone [51]. A recent study showed that the *Arabidopsis thaliana* mutant chs5 remoulded the profile of isoprenoid-derived metabolites (including monoterpenoids and sesquiterpenoids), which further impacted the specific recruitment of *Streptomyces* into the microbiota [52]. SA, a key immune regulator, also modulates colonization of the root microbiome by specific bacterial taxa, including *Streptomyces* and *Bacillus* [53]. A previous study also revealed that the root colonization of *Streptomyces* strains was increased in cpr5 mutant plants, which constitutively produce salicylate, indicating that SA mediated the interaction of the host plant and *Streptomyces* strains in the root zone. However, *Streptomyces* species are not directly attracted by the presence of SA in root exudates and soil [54]. The mechanism by which plants recruit these microbial groups needs to be further verified.

By performing SynCom experiments based on the bacterial strains derived from rhizosphere soil and roots, the microbial effects on the monoterpene accumulation of citrus in the core region were further verified. Six of seven monoterpenes showed high contents in core regions, four of which could be promoted by *Streptomyces* strain Strep-4 and its SynCom. We also found that the *Streptomyces* strain and SynCom activated terpene synthesis and boosted the accumulation of monoterpenes in the leaves of *C. reticulata* ‘Chachi’ in an immune-dependent manner. Monoterpenes act as microbe-inducible plant volatiles, which, as part of plant-derived volatile blends, have the potential to enhance plant innate immune signalling [55, 56].

Numerous MAGs enriched in core region soil contained terpene BGCs, and the abundance of terpene clusters was higher in the core region than in the non-core region. We also verified that soil microbes with the potential for terpene synthesis could enter the root. First, the 16S rRNA genes of 14 MAGs closely matched 13 OTUs from root endophytes, some of which also showed genomic potential for terpene biosynthesis. Second, 12 endophyte OTUs were mapped to the cultivated strains derived from the rhizosphere soil. These findings also implied that endophyte microbes could enter the root from rhizosphere soil and facilitate the synthesis of terpenes in citrus. Finally, the expression of microbial terpene BGCs could be detected in the RNA-seq data of the roots. TEM observation validated that endophyte microorganisms exist in the root cells and take part in lipid droplet synthesis. Therefore, we concluded that the microorganisms entered roots from rhizosphere soil and facilitated the production of monoterpenes in the citrus plants.

## Conclusions

**Fig. 7:**
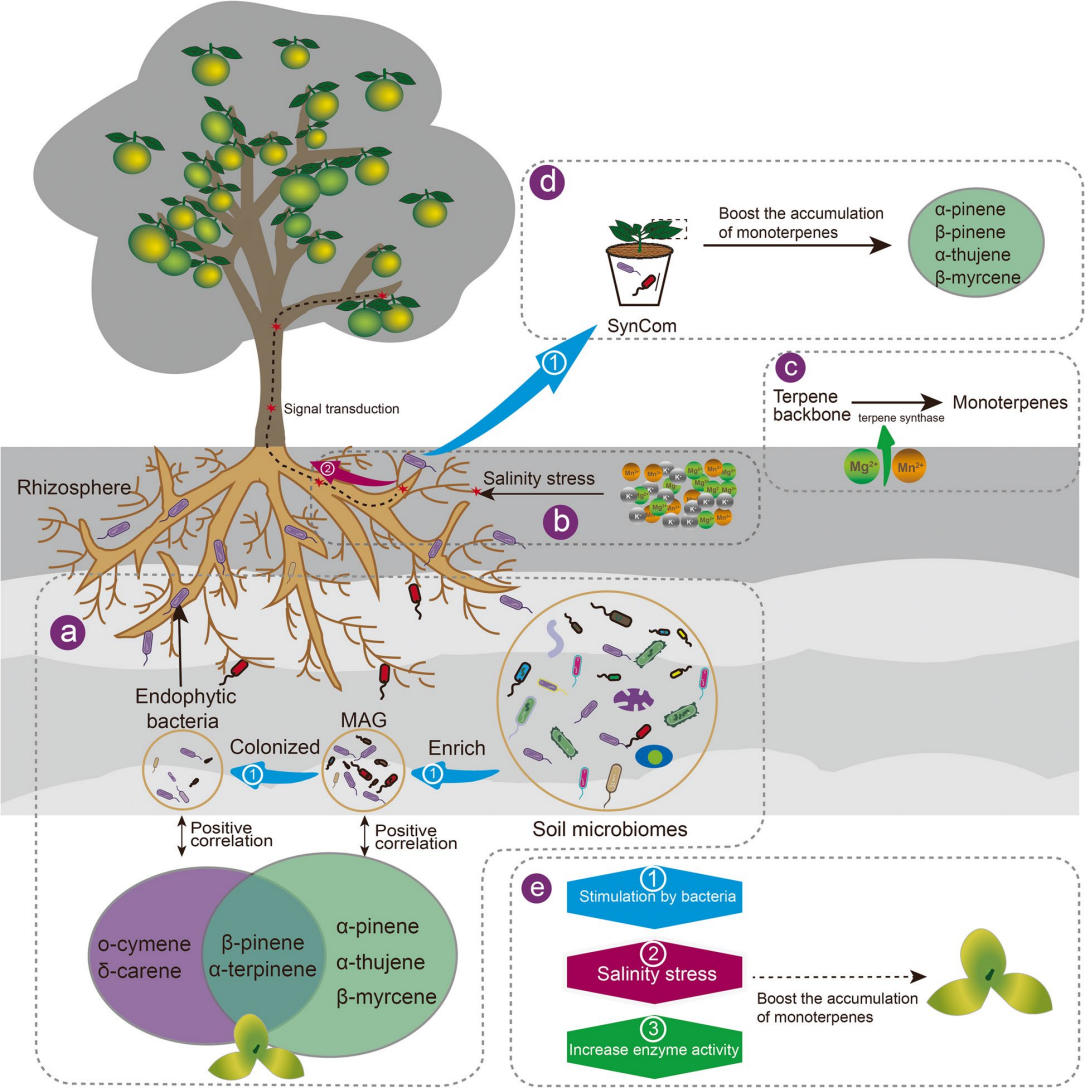
Mechanisms underlying soil chemical properties and microorganism-mediated effects on the accumulation of monoterpenes. **a** Rhizosphere soil was enriched in salinity-tolerant microorganisms with the capacity for terpene biosynthesis. **b**, **c** A special soil environment (high salinity, Mg, Mn and K) enhances the monoterpenoid content by promoting the expression of salt stress-responsive genes and activities of terpene backbone synthase. **d** SynCom boosts the accumulation of monoterpenes in the leaves of *C. reticulata* ‘Chachi’ in an immune-dependent manner. **e** We assumed that the rhizosphere soil and endophyte bacteria co-contributed to the accumulation of monoterpenes by triggering the plant immune response or providing intermediate products for the plant. MAGs: metagenome-assembled genomes

Using multi-omics approaches, we were able to identify the factors that contributed to the increased concentration of monoterpenes in the citrus peel in core regions. The special features of soil, including higher contents of micro-elements, Mg and EC_sw_, were positively correlated with monoterpene biosynthesis. Rhizosphere soil and endophyte microorganisms co-contributed to the accumulation of monoterpenes. Synthetic community assays verified that *Streptomyces* strain Strep-4 and its SynCom derived from the core region can boost the accumulation of monoterpenes in the leaves of *C. reticulata* ‘Chachi’. We also proposed that rhizosphere microorganisms might activate terpene synthesis and regulate monoterpene accumulation through interactions with the host immune system. This study clearly illustrated the effects of soil properties and soil microorganisms on monoterpene production in the citrus peel of plants from core regions (Fig. 7). The results provide a fundamental basis for improving fruit quality depending on bioactive components by formula fertilization and precise microbiome management in agricultural practice.

## Material and methods

### Sample collection

Three representative orchards (named TianMa, Wubao and Maichong) with prominent *C. reticulata* ‘Chachi’ production in Xinhui County (geo-authentic product region) of Guangdong Province were selected as the core region (detailed information can be found in Table S1). Four orchards (non-geo-authentic product region) far from the core region were selected to represent the non-core region (Table S1). Approximately six to thirteen healthy citrus trees with ages ranging from 8 to 10 years in each orchard were selected for sampling. For each tree, rhizosphere soil, bulk soil and leaf, root and fruit samples were collected. First, the rhizosphere soil and root samples were collected from the four ordinate directions approximately 1 m away from the trunk. After removing the topsoil using a shovel, roots from a depth of 10–15 cm were collected. Then, the soil that was not tightly attached to the roots was gently removed, and the roots were pooled into 15-ml sterile tubes and transferred to the laboratory on dry ice. In the laboratory, the roots were cut into small pieces and added to 15-ml tubes containing 10 ml of sterile PBS-S buffer. Then, the roots were washed on a shaking platform for 20 min at 25 r/s to remove soil that tightly adhered to the root surface. The soil that was washed from the roots was transferred to a 50-ml Falcon tube and collected as the rhizosphere soil sample. After the ultrasound treatments, the root pieces were sterilized with 75% alcohol for 1 min and washed with sterile water 5 times. Then, clean roots were collected to extract the DNA of the endophyte microbiome using the CTAB method [57]. Bulk soil was collected from sites without plants and near the selected trees. Then, the bulk soil samples were immediately frozen on dry ice and transferred to the laboratory. In total, we obtained 58 rhizosphere soil samples and 37 bulk soil samples from seven representative orchards. Second, ripened fruits of similar size (6 cm diameter), the mature leaves closest to the fruit and the roots from the tree were also sampled, frozen in liquid nitrogen immediately and stored at − 80 °C. A total of 167 samples from peels, leaves and roots were harvested for RNA-seq analysis.

### Extraction of essential oil and gas chromatography analysis

After the fruit peel was smashed, 15 g of peel powder was hydrated with 300 ml of distilled water in a standard extractor for extracting essential oil for 3 h. Then, the essential oil was dried over anhydrous sodium sulphate until all the water was dried and stored in a dark glass bottle at 4 °C. Gas chromatography–mass spectrometry (GC–MS) analyses were carried out using a Shimadzu GCMS-QP2010 Ultra spectrometer.

### Comparative analyses of essential oils

The difference in essential oil content in citrus peel between the two regions was detected using the Wilcoxon rank sum test with an adjusted *P* value of < 0.05. To link essential oil content with citrus planting area, leave-one-out cross validation (LOOCV) with the random forest algorithm was performed based on all 56 samples (R package: randomForest). We used 55 samples as a training set to perform feature selection and build a random forest classifier; then, we used the classifier to predict the remaining sample and recorded the feature importance rank. After each sample had been tested, seven monoterpenes were selected as markers according to the importance of features.

### Measurement of soil chemical properties

The physical and chemical properties of the soil were measured from soil samples harvested in each field. The organic matter was determined by the oven heating method. For pH analysis, soils were extracted in deionized water for one hour to achieve a soil:solution ratio of 1:2.5. The water pH was subsequently measured using a combination pH electrode. To measure the electrical conductivity of soil water (EC_sw1:5_), soils were extracted in deionized water for 1 h to achieve a soil:water ratio of 1:5. The EC_sw1:5_ of the extract was subsequently measured using a conductivity metre. The available N, P and K in soil were determined according to the previous literature [58]. Soil micronutrient concentrations were measured using the diethylenetriaminepentaacetic acid (DTPA) extraction method [52]. A total of 10 g of air-dried soil was mixed with 20 ml of DTPA extraction solution (0.005 M DTPA, 0.01 M CaCl_2_, 0.1 M triethanolamine, pH 7.3) by shaking for 2 h at room temperature. The liquid supernatants were filtered and subsequently analysed by inductively coupled plasma–optical emission spectrometry (ICP–OES, Optima 7300 DV, PerkinElmer) for Fe, Mn, Cu, Zn and Mg concentrations. Soil properties from the two regions were compared using the Wilcoxon rank sum test with an adjusted *P* value of < 0.05.

### RNA extraction, transcriptome sequencing and analysis

RNA from the leaves, roots and peels was extracted using a Column Plant RNAout Kit according to the manufacturer’s instructions (TIANDZ, China). The extracted RNA was quantified and assessed for integrity using a NanoDrop (Thermo, USA) and 2100 Agilent Bioanalyzer (Agilent, USA) prior to subsequent experiments. One microgram of qualified RNA from each sample was used to construct the BGI-based mRNA-seq library as described in a previous study [59]. Then, the library was sequenced on the BGISEQ platform using a 100-bp pair-ended strategy.

The raw reads were filtered by SOAPfilter, an application included in the SOAPdenovo package [60]. An average of 6 Gb of high-quality data were generated for each sample. Then, the resulting high-quality reads from each sample were mapped against the *Citrus sinensis* reference genome [35] using HISAT2 (version 2.0.4) [61]. FeatureCounts from the Subread package (version 1.6.4) [62] was used to count the reads that mapped to each of the protein-coding sequences. The expression level of each gene in each sample was calculated as the FPKM with StringTie2 [63]. The differentially expressed genes were identified using DESeq2 (version 1.6.4) [64] based on the read matrix, with an FDR-corrected *P* value < 0.05 and absolute fold change value > 2. The enrichment of differentially expressed genes in pathways was detected using Fisher’s exact test.

The β-pinene synthase sequence (Q8L5K2) was downloaded from *Citrus medica* var*. limon* in UniProt [65] as a reference sequence, and the β-pinene synthase gene in our data was identified by a BLASTP search. Then, we filtered the hits according to the following criteria: identity ≥ 85, *E* value ≤ 1e − 5 and bit score ≥ 65. β-Pinene synthesis gene profiling was performed by summing up the expression of genes blasted out. The promoter sequence of the *DXS* gene was extracted, and the transcription factor DNA-binding preference was predicted by JASPAR [66].

### Microbial DNA extraction and de novo assembly

The microbial DNA of rhizosphere soil and bulk soil samples was extracted using a PowerSoil DNA isolation kit (Mobio Labs, Inc., Solana Beach, CA, USA) according to the manufacturer’s protocol. All DNA samples were sequenced on the BGISEQ platform using a 100 bp paired-end sequencing strategy. More than 2.34 Tb of shotgun metagenomic sequences were generated, yielding an average of 26 Gb for each sample.

All the raw data were filtered by SOAPnuke (v1.5.6) [67] by removing adaptor sequences and trimming low-quality reads. Ninety-two samples were separated into twelve groups based on their microbial community similarity as calculated by Mash [68]. All the metagenomic reads from each group were pooled together to perform de novo assembly using Megahit (version 1.0.3) [69] with the meta-large preset parameter. A total of 248 M assembled contigs were generated with a total length of 160 Gb. Then, all clean reads were mapped against the contigs to calculate the read utilization rate. An average of 43.3% of the total reads were used to construct the contigs.

### Gene profile, functional profile and taxonomic profile

Genes from the metagenome were predicted across the contigs with a length of > 500 bp by Prodigal [70] using meta mode, resulting in approximately 167,559,564 original genes. A unique gene set was constructed using an amino acid sequence by Cd-hit [71] with the same coverage parameter of 0.9 and identity cut-off of 95% as described in a previously published paper [72]. Finally, a nonredundant gene set including 93,588,611 genes was generated with a total length of more than 57.96 Gb.

Gene functions were annotated by aligning protein sequences of nonredundant genes against KEGG (version 81) [73] using Diamond [74], and the blast results meeting the criterion “hit score > 60, win score = 1” were retained. Taxonomic annotations were implemented by mapping genes to the NCBI Non-Redundant Protein Sequence Database with Diamond, and the taxonomic classifications of each gene were determined by filtering the alignments. For KO abundance calculation, the filtered reads were mapped to the nonredundant gene set by Bowtie2 [75] with default parameters. The absolute abundance of each gene was calculated as described in a previous study [76]. The KO profile and taxonomic profile were generated by summing the absolute abundances of genes affiliated with the same KO and the same taxon, respectively.

### Comparative analysis of the functionalities of rhizosphere soil microbiomes

The absolute abundance of each KO in the sample was normalized by dividing by the sum of all selected KO abundances. The differentially enriched KOs were identified based on normalized abundance using the Wilcoxon rank sum test with an adjusted *P* value of < 0.05. Then, the differentially enriched KEGG pathways were identified as described in a previous study [77]. KEGG pathways with a reporter score (|*z* score|) of > 1.7 could be considered significantly differentiated pathways.

### MAG reconstruction and bacterial biosynthesis gene cluster analysis

Contigs longer than 2 kb were clustered to reconstruct metagenome assembly genomes (MAGs) using differential coverage binners such as MaxBin2 [78], CONCOCT [79] and MetaBAT [80]. Subsequently, CheckM [81] was used to evaluate the qualities of MAGs. A total of 6053 draft genomes were obtained by filtering with a threshold of > 70% completeness and < 10% contamination or > 50% completeness and < 5% contamination. After dereplicating at 98% nucleotide identity using dRep [82], a final set that included 3350 nonredundant MAGs was obtained. The taxonomic assignment of these MAGs was performed using Genome Taxonomy Database Toolkit (GTDB-Tk) (version 1.3.0) [83] with the GTDB Release 95 taxonomy [84]. Then, we mapped the clean reads of each sample to the 3350 bins using Bowtie2 to obtain the final abundance of each bin.

We predicted the biosynthetic gene clusters (BGCs) by using antiSMASH5.0 [85] based on contigs larger than 10 kb. To precisely identify the products, the predicted BGCs were aligned with those reported in the MIBiG [86] repository using BLASTP. The closest homologue cluster was selected based on the highest cumulative BLAST score. The BGC profile was generated by BiG-MAP, which is an automated pipeline for profiling metabolic gene cluster abundance and expression in microbiomes [43].

### 16S rRNA gene amplicon sequencing of the endophyte microbiome

The V4 region of the bacterial 16S rRNA gene was amplified from the root samples with primers 515F (5′-GTGCCAGCMGCCGCGGTAA-3′) and 806R (5′-GGACTACHVGGGTWTCTAAT-3′) and sequenced on the BGISEQ platform. The raw reads were filtered by SOAPnuke as mentioned above. Over 0.2 million reads were obtained in every sample after filtering. For every sample, we subsample 50 thousand reads for downstream analysis. The representative sequences were selected by UPARSE [87], which resulted in a total of 2491 OTUs. The original reads were assigned back to their OTUs using USEARCH [88]. To obtain the taxonomic information of the OTUs, representative sequences of each OTU were aligned against the RDP database [89]. The OTUs and sequences, which were defined as unknown, chloroplast, mitochondria or plants, were removed. The taxonomic dissimilarity analysis between samples was performed using the PCoA method based on unweighted UniFrac distances (beta diversity). The OTUs differentially enriched between core and non-core samples were identified with DESeq2 [64]. *P* values were corrected for multiple testing using the FDR method in DESeq2. All items with corrected *P* values ≤ 0.05 and an absolute fold change > 1.2 were considered significant.

### Correlation-based network

For each network, Spearman correlation scores were calculated using *cor.test* [90]. *P* values were corrected for multiple testing using the FDR [91] method, and the results were filtered with FDR-corrected *P* values ≤ 0.05 and an absolute correlation R ≥ 0.5. The results were visualized in a graph displaying the correlation R and *P* values using Cytoscape (version 3.7.0) [92].

### Bacterial strains isolated from rhizosphere soil and the endophyte of citrus

The rhizosphere soil of the *C. reticulata* ‘Chachi’ planted in the Maichong orchard was collected, and 10 g was diluted in 100 ml of PBS buffer and fully mixed on a shaking platform for 10 min at 25 r/s. The mixed buffer was then subjected to centrifugation for 5 min at 200 × g. The supernatants were diluted from 10^−1^ to 10^−7^, and then 10^−4^ and 10^−6^ dilutions were distributed and cultivated in Luria–Bertani medium and Gauze’s synthetic medium No. 1 for 48–72 h at 28 °C. Of these, Gauze’s synthetic medium No. 1 was used for Actinomycetes strain isolation, and Luria–Bertani medium was used to isolate other bacterial strains. After purification by three consecutive platings on solidified media, 94 bacterial clones were obtained.

The roots of *C. reticulata* ‘Chachi’ collected from the Maichong orchard were sterilized by ultrasound treatments and 75% alcohol as described above. The root pieces were added into 15-ml sterile tubes with PBS buffer and then smashed. After centrifugation, the supernatant was spread on Luria–Bertani medium and Gauze’s synthetic medium No. 1 after dilution. After growth for 24 h at 30 °C, single colonies were selected. After subculturing three times, each colony was retained for subsequent experiments.

The V4 region of the bacterial 16S rRNA gene was amplified as described above. The barcoded PCR product for each strain was sequenced on the BGISEQ platform. The representative stain of the endophyte OTU was identified with the best hit of the 16S rRNA gene with a gene similarity of more than 97%, resulting in 35 OTUs with a representative strain.

### Synthetic community assay

To validate the promoting effect of marker microbes on *C. reticulata* ‘Chachi’ monoterpene accumulation, SynCom experiments were conducted based on three soil-derived strains (Strep-4 from *Streptomyces*, Steno-6 from *Stenotrophomonas* and Pseud-1 from *Pseudomonas*) and one endophyte strain, Serra-11, from *Serratia*. Surface-sterilized seeds of *C. reticulata* ‘Chachi’ were germinated in a pot with sterilized nursery media for 60 days. The seedlings were then transferred into a pot with sterilized field soil. When the citrus seedlings reached 5 cm, the SynCom assay was carried out. The optical density (OD600) of the strains was adjusted to 0.5, and bacterial cells were collected by centrifugation at 5000 rpm for 10 min. The bacterial cells were resuspended in 20 ml of sterile water, and the bacterial suspension was immediately added to the roots of the plants. The control group was treated with sterile water.

After 30 days of co-culture, citrus leaves were collected to extract the essential oils. Leaves for essential oil extraction were crushed, and 600 mg of powders was weighed and extracted with 3.0 ml of normal hexane for 30 min at 60 °C. The supernatant was aspirated after centrifugation for 10 min at 10,000 rpm. The essential oil was filtered via a 0.2-µm filter before performing GC–MS. At the same time, RNA was extracted from the leaves to perform RNA sequencing on the BGISEQ platform.

### Bacterial genome sequencing and assembly

Twelve bacterial strains were sequenced on the BGISEQ platform to investigate their potential to produce monoterpenes, including three soil-derived strains (Strep-4, Steno-6 and Pseud-1) and nine endophyte strains (Flex-rb329, Adve-rb203, Pseu-rb243, Pseu-rb338, Sten-rb415, Pseu-rb421, Sten-rb436, Burk-rb455 and Serra-11). The 100 bp reads were de novo assembled by SPAdes (version 3.15.3) [93]. After assembly, we found that eight endophyte strains were contaminated, excluding Serra-11. Genes were predicted by prodigal [70] and functions were annotated using Diamond [74] as described above.

### Sterile root preparation

The seeds of *C. reticulata* ‘Chachi’ were soaked in 1 mol/L NaOH solution for 1–3 h to remove the pectin on the surface and soften the seed coat. Then, each seed was rinsed under running water and transferred to the benchtop. The inner and outer seed coats were removed under a dissecting microscope, and the coatless seeds were soaked in 0.5% sodium hypochlorite solution (adding 0.1% Tween 20) for 10 min. Then, seed embryos were washed with sterile water 3 times and inoculated onto MS culture medium for 50 days. The mature area was fixed when the diameter of the main root reached 0.5–2 mm (histological and cytological operations can be performed according to the method above).

### Gram staining and transmission electron microscopy (TEM)

Normally growing juvenile roots of *C. reticulata* ‘Chachi’ in Xinhui were divided into small blocks (at 0.5 mm length), fixed in 4% paraformaldehyde and 0.5% glutaraldehyde (0.1 M PBS, pH 7.2) and post-fixed in 1% osmium for 2 h at 25 °C. After a series of alcohol dehydrations, samples were embedded in Epon 812 epoxy resin. The sections were cut into 1-μm sections by a Leica RM2155 instrument (Leica, Germany). According to the Gram staining kit (G1060, Beijing Solaibo Technology Co., Ltd., Beijing), the sections were stained with ammonium oxalate crystal purple for 1 min, rinsed with distilled water, dyed with iodine solution for approximately 1 min, washed with distilled water, decolorized for 40 s with decolorizing solution, stained with safranine for 1 min, washed with distilled water, sealed under slides and observed under a Lecia DM6 microscope (Leica, Germany).

For TEM observation, the above embedded blocks were cut by a Leica UC7 (Leica, Germany) with a thickness of 60–90 nm. After samples were stained with uranium dioxide acetate and lead citrate, they were observed and photographed under a Philip Fei-Tecnai 12 transmission electron microscope.

### Quantitative real-time PCR analyses

Total RNA of leaves was extracted using the Column Plant RNAout Kit according to the manufacturer’s instructions (TIANDZ, China). The extracted RNA was quantified and assessed for integrity using a NanoDrop spectrophotometer (Thermo, USA) and reverse transcribed into cDNA using the PrimeScript RT Reagent Kit (TaKaRa, Dalian, China). The β-actin gene (forwards primer: 5′-CACACTGGAGTGATGGTTGG-3′, reverse primer: 5′-ATTGGCCTTGGGGTTAAGAG-3′) was used as a housekeeping gene. The primers for the *DXS* gene (forwards primer: 5′-GGCTCTCTCTTCCGGGATTG-3′, reverse primer: 5′-TCACCATCTTCGTCGCTTCC-3′) were designed using PRIMER (version 6.0). The expression levels of *DXS* were measured by quantitative real-time PCR using SYBR Green Supermix on the CFX96 Real-Time PCR Detection System (BIO-RAD) according to the manufacturer’s protocol. Each sample had three biological replicates, with three technical replicates for each biological replicate. The relative expression level was normalized by using the 2^−ΔΔCT^ method [94].

## Supplementary Information

**Additional file 1: Table S1.** Summary of sampled orchards in Guangdong and Guangxi provinces of China. **Table S2.** Contents of essential oils in peel samples from different orchards. **Table S3.** Highly expressed genes from the terpenoid backbone pathway in root, leaf, and peel samples from the core region. **Table S4.** Correlation between monoterpenes and highly expressed KOs from roots, leaves and peels. **Table S5.** Soil physical and chemical properties of rhizosphere soil samples. **Table S6.** Phylum composition of all metagenome samples (BS represents bulk soil, and RS represents rhizosphere soil). **Table S7.** Genus composition of all metagenome samples (BS represents bulk soil, and RS represents rhizosphere soil). **Table S8.** Detailed information on 3350 metagenome-assembly genomes (MAGs). **Table S9.** MAG genera differentially enriched between the core and non-core regions. **Table S10.** Isolation of soil- and root-derived bacteria. Table S11. Genome information of bacterial strains. **Table S11.** Highly expressed genes detected in *C. reticulata *‘Chachi’ leaves after inoculation with Strep-4 and its SynComs. **Table S12.** Genome information of the bacterial strain. **Table S13.** Differentially enriched endophyte microbial OTUs in the core and non-core regions. **Table S14.** Correlation between monoterpenes and endophyte microbial OTUs enriched in the core region. **Table S15.** The similarities of the MAG 16S rRNA gene and endophyte OTU sequences.

**Additional file 2: Figure S1.** Differentially expressed genes in leaf, peel and root samples between the two regions. (a) The numbers in the figure represent the number of differentially expressed genes. (b) FPKM of 1-deoxy-D-xylulose-5-phosphate synthase (*DXS*) between regions. Statistical differences in peel, leaves, and roots between the two regions were evaluated by the Wilcoxon rank sum test. (c) Relative expression of *DXS* between regions was measured using qRT–PCR. Statistical differences in peel, leaves, and roots between the two regions were evaluated by the Wilcoxon rank sum test. **Figure S2.** Correlation network of transcript KOs and monoterpenes. The correlation-based network between highly expressed genes in the leaves (a) and peels (b) (nodes) and monoterpenes (triangles). Node size corresponds to the degree of each monoterpene. The thickness and colour of the edges denote the strength and significance, respectively. Solid and dashed edges indicate positive and negative correlations, respectively. **Figure S3.** The taxonomic composition of the rhizosphere soil microbiome at the phylum level. Only the microbial phyla with the top 10 relative abundances among bacteria (a) and archaeal phyla (b) are shown. **Figure S4.** Microbial composition of the root-associated microbiome and its relationship to soil chemical properties. (a) PCoA based on the genus abundance profile was performed to assess the influences of geographical location and microhabitat on microbial communities. (b) Pairwise comparisons of environmental factors are shown, with a colour gradient denoting Spearman’s correlation coefficient. Taxonomic (endophyte and metagenomes) and functional composition relationships with each environmental factor were detected by partial Mantel tests. Edge width corresponds to Mantel’s R statistic for the corresponding distance correlations, and edge colour denotes the statistical significance based on 9,999 permutations. Solid and dashed edges indicate positive and negative correlations, respectively. **Figure S5.** The correlation-based network between highly expressed genes (nodes) in the root and MAGs positively correlated with monoterpenes (triangles). Node size corresponds to the degree of each genus. The thickness and colour of the edges denote the strength and significance, respectively. Solid and dashed edges indicate positive and negative correlations, respectively. **Figure S6.** Information on secondary metabolite BGCs in newly reconstructed MAGs. Distribution of different biosynthetic gene cluster (BGC) compositions in phyla. **Figure S7.** The abundance of terpene BGCs in the microbial community shifted between the core region and non-core region. **Figure S8.** PCoA based on the endophyte OTU profile was performed to assess the influences of geographical location. **Figure S9.** The endophyte OTUs annotated to the genera enriched in the rhizosphere soil of the core region. The left boxplot represents the relative abundances of genera enriched in rhizosphere soil samples from the core region, and the right boxplot represents the relative abundances of endophyte OTUs in the core region. **Figure S10.** The genome of the endophyte strain Serra-11. (a) Circular representation of the genome of *Serratia marcescens* strain rb-red1. The inner circle shows the 103 contigs sorted by size. The middle circle shows GC skew. The outer circle shows the functional genes annotated to the terpene backbone synthesis pathway. (b) Synthesis process of monoterpenoid precursors. The blue circle represents the substrate or product, and the orange box represents enzymes. **Figure S11.** The expression of microbial biosynthetic gene clusters in citrus peel, leaf and root samples. (a) The number of microbial biosynthetic gene clusters found in citrus peel, leaf and root samples. (b) The expression of biosynthetic gene clusters in citrus peel, leaf and root samples. **Figure S12.** Microscopic observation of root endophytes. (a-f) Microstructure of root endophytic bacteria in *C. reticulata* ‘Chachi’; a, c and e show the overall view of the root in *C. reticulata* ‘Chachi’; b, d and f show the local enlargement of the root in *C. reticulata *‘Chachi’. b is the local magnification in the black box in a. Gram-positive bacteria were distributed in the root cortical cells (black arrow). d shows local magnification of the black box area in c. Gram-positive bacteria were distributed in the intercellular space of root cortex cells (black arrows). f shows local magnification of the black box area in e. Gram-negative bacteria were distributed in the root cortical cells (black arrows). Bar = 50 μm for a, c and e. Bar = 25 μm for b, d and f. (g-i) Ultrastructure of root endophytic bacteria in *C. reticulata* ‘Chachi’ detected by TEM; (g) Endophytic bacteria in the intercellular space of the root cortex in *C. reticulata* ‘Chachi’. The white arrows indicate the endophytic bacteria. Bar = 1 μm. (h) Endophytic bacteria in the cells of the root cortex in *C. reticulata* ‘Chachi’. The white arrows indicate endophytic bacteria. Bar = 1 μm. (i-l) Endophytic bacteria in developing cells of the root vascular cylinder in *C. reticulata* ‘Chachi’. The white arrow shows the black osmiophilic droplets in the bacterial membrane and the intimal structure of the bacterium, and the black arrow shows the grey osmiophilic droplets in the cytoplasm of the bacterium. Bar = 500 nm. IS: intercellular space. (m, n). Ultrastructure of the root of aseptic seed culture in *C. reticulata *‘Chachi’. No endophytic bacteria occurred in the intercellular space and cytoplasm of the root cortical cells (o) and root vascular cylinder cells (p) in *C. reticulata* ‘Chachi’. Bar = 2 μm. M: mitochondrion. G: Golgi apparatus; S: starch; N: nucleus; ER: endoplasmic reticulum. **Figure S13.** The Sudan black dye lipid droplet method shows the distribution of lipid droplets in the root and peel**.** (a-c) Few lipid droplets in the vascular cylinder and cortical cells of aseptic culture plants produced by seeds. (d-i) Abundant lipid droplets in the vascular cylinder and cortical cells of young roots (d-f) and more mature roots (g-i) collected from the core region. (j-l) Vascular bundles of the peels collected from the core region. j provides an overall view of the peel with two secretary cavities and a vascular bundle. k and l are longitudinal sections and cross-sections of the vascular bundle with lipid droplets, respectively. Black arrows indicate lipid droplets.

## Data Availability

The data that support the findings of this study have been deposited into the CNGB Sequence Archive (CNSA) of the China National GeneBank Database (CNGBdb) under accession number CNP0002028.

## Abbreviations

*FLS2*: LRR receptor-like serine/threonine-protein kinase FLS2
*WRKY1*: WRKY transcription factor 1
*MPK3*: Mitogen-activated protein kinase 3
*MKS1*: MAP kinase substrate 1
*OXI1*: Serine/threonine-protein kinase OXI1
*RPS2*: Disease resistance protein RPS2
*RPM1*: Disease resistance protein RPM1
*PR1*: Pathogenesis-related protein 1
*WRKY29*: WRKY transcription factor 29
*SUMM2*: NB-LRR protein SUMM2
*VSP2*: Vegetative storage protein 2
*PYR/PYL*: Abscisic acid receptor PYR/PYL family
NRPS: Non-ribosomal peptide synthetase
T1PKS: Type I polyketide synthase
MAPK: Mitogen-activated protein kinase
FPKM: Fragments per kilobase of exon per million mapped fragments
