# Supplementary figures and images for: Soil conditions and the plant microbiome boost the accumulation of monoterpenes in the fruit of Citrus reticulata ‘Chachi’
Source: Microbiome. 2023 Mar 28;11:61. doi: 10.1186/s40168-023-01504-2 (PMC10044787; doi:10.1186/s40168-023-01504-2)

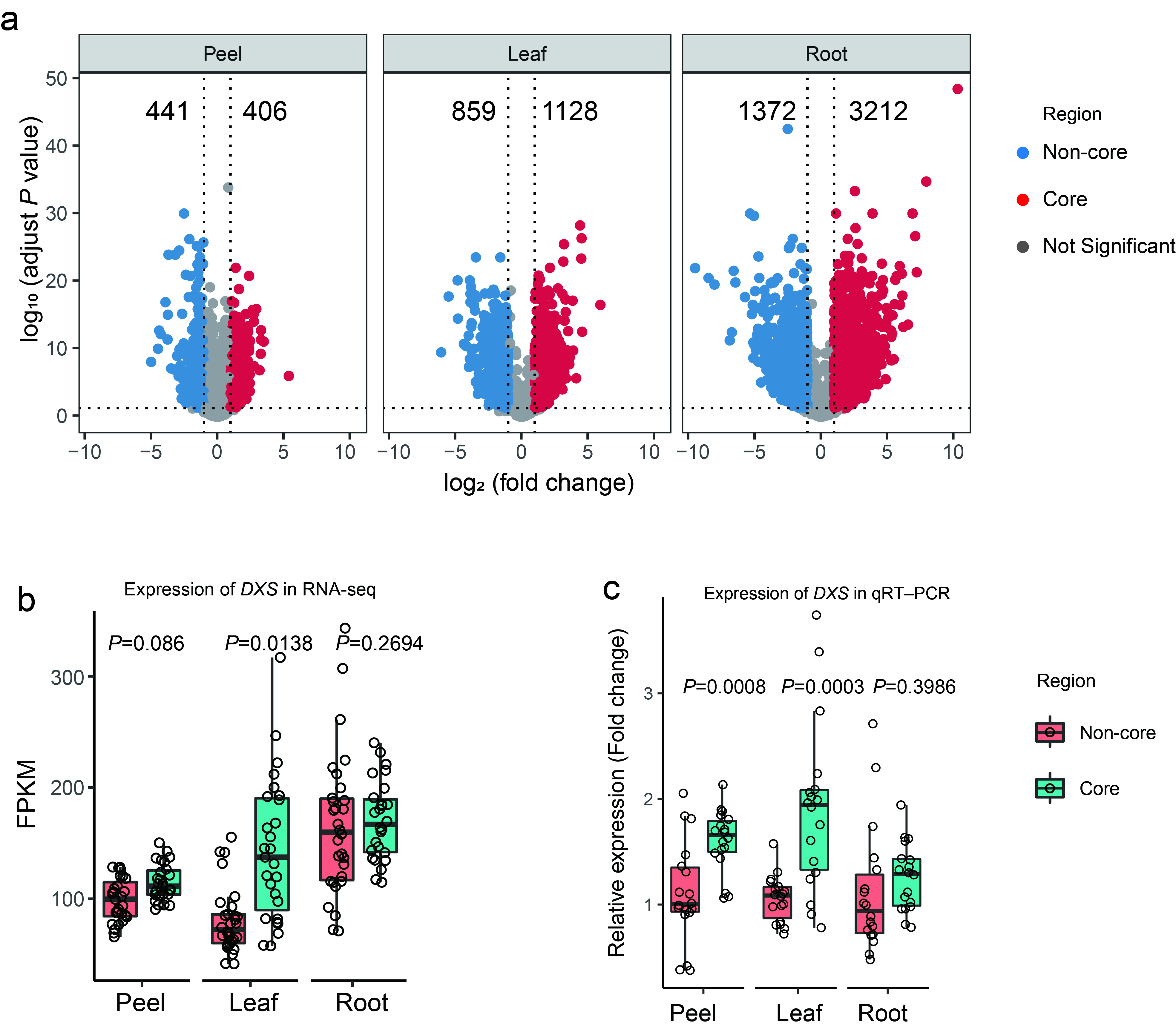

Supplement: Supplementary file 3 — Additional file 2: Figure S1. Differentially expressed genes in leaf, peel and root samples between the two regions. (a) The numbers in the figure represent the number of differentially expressed genes. (b) FPKM of 1-deoxy-D-xylulose-5-phosphate synthase (DXS) between regions. Statistical differences in peel, leaves, and roots between the two regions were evaluated by the Wilcoxon rank sum test. (c) Relative expression of DXS between regions was measured using qRT–PCR. Statistical differences in peel, leaves, and roots between the two regions were evaluated by the Wilcoxon rank sum test. Figure S2. Correlation network of transcript KOs and monoterpenes. The correlation-based network between highly expressed genes in the leaves (a) and peels (b) (nodes) and monoterpenes (triangles). Node size corresponds to the degree of each monoterpene. The thickness and colour of the edges denote the strength and significance, respectively. Solid and dashed edges indicate positive and negative correlations, respectively. Figure S3. The taxonomic composition of the rhizosphere soil microbiome at the phylum level. Only the microbial phyla with the top 10 relative abundances among bacteria (a) and archaeal phyla (b) are shown. Figure S4. Microbial composition of the root-associated microbiome and its relationship to soil chemical properties. (a) PCoA based on the genus abundance profile was performed to assess the influences of geographical location and microhabitat on microbial communities. (b) Pairwise comparisons of environmental factors are shown, with a colour gradient denoting Spearman’s correlation coefficient. Taxonomic (endophyte and metagenomes) and functional composition relationships with each environmental factor were detected by partial Mantel tests. Edge width corresponds to Mantel’s R statistic for the corresponding distance correlations, and edge colour denotes the statistical significance based on 9,999 permutations. Solid and dashed edges indicate positive and nega [file 40168_2023_1504_MOESM2_ESM.zip › Supplementary Figures/Figure S1.jpg]

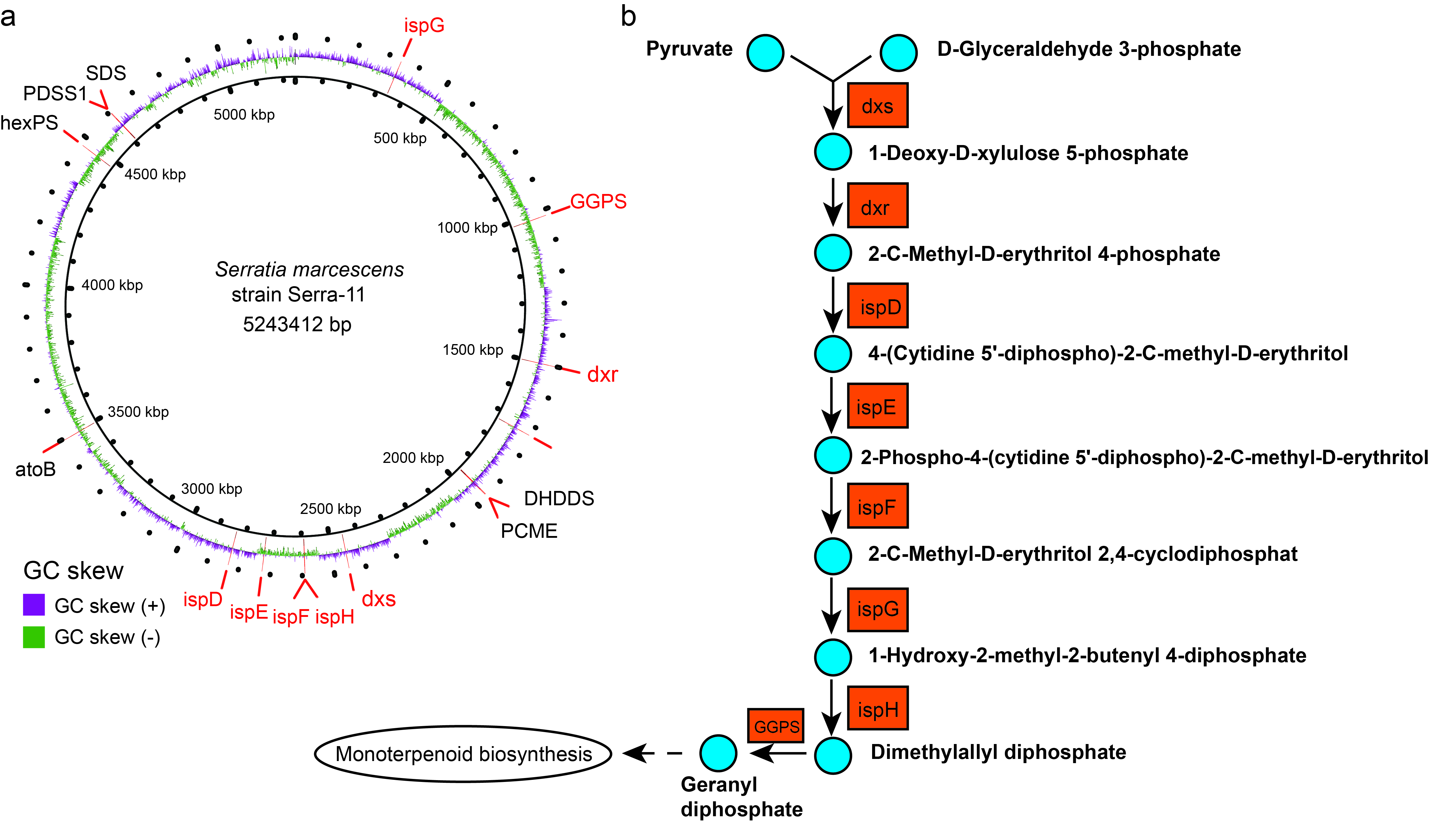

Supplement: Supplementary file 3 — Additional file 2: Figure S1. Differentially expressed genes in leaf, peel and root samples between the two regions. (a) The numbers in the figure represent the number of differentially expressed genes. (b) FPKM of 1-deoxy-D-xylulose-5-phosphate synthase (DXS) between regions. Statistical differences in peel, leaves, and roots between the two regions were evaluated by the Wilcoxon rank sum test. (c) Relative expression of DXS between regions was measured using qRT–PCR. Statistical differences in peel, leaves, and roots between the two regions were evaluated by the Wilcoxon rank sum test. Figure S2. Correlation network of transcript KOs and monoterpenes. The correlation-based network between highly expressed genes in the leaves (a) and peels (b) (nodes) and monoterpenes (triangles). Node size corresponds to the degree of each monoterpene. The thickness and colour of the edges denote the strength and significance, respectively. Solid and dashed edges indicate positive and negative correlations, respectively. Figure S3. The taxonomic composition of the rhizosphere soil microbiome at the phylum level. Only the microbial phyla with the top 10 relative abundances among bacteria (a) and archaeal phyla (b) are shown. Figure S4. Microbial composition of the root-associated microbiome and its relationship to soil chemical properties. (a) PCoA based on the genus abundance profile was performed to assess the influences of geographical location and microhabitat on microbial communities. (b) Pairwise comparisons of environmental factors are shown, with a colour gradient denoting Spearman’s correlation coefficient. Taxonomic (endophyte and metagenomes) and functional composition relationships with each environmental factor were detected by partial Mantel tests. Edge width corresponds to Mantel’s R statistic for the corresponding distance correlations, and edge colour denotes the statistical significance based on 9,999 permutations. Solid and dashed edges indicate positive and nega [file 40168_2023_1504_MOESM2_ESM.zip › Supplementary Figures/Figure S10.jpg]

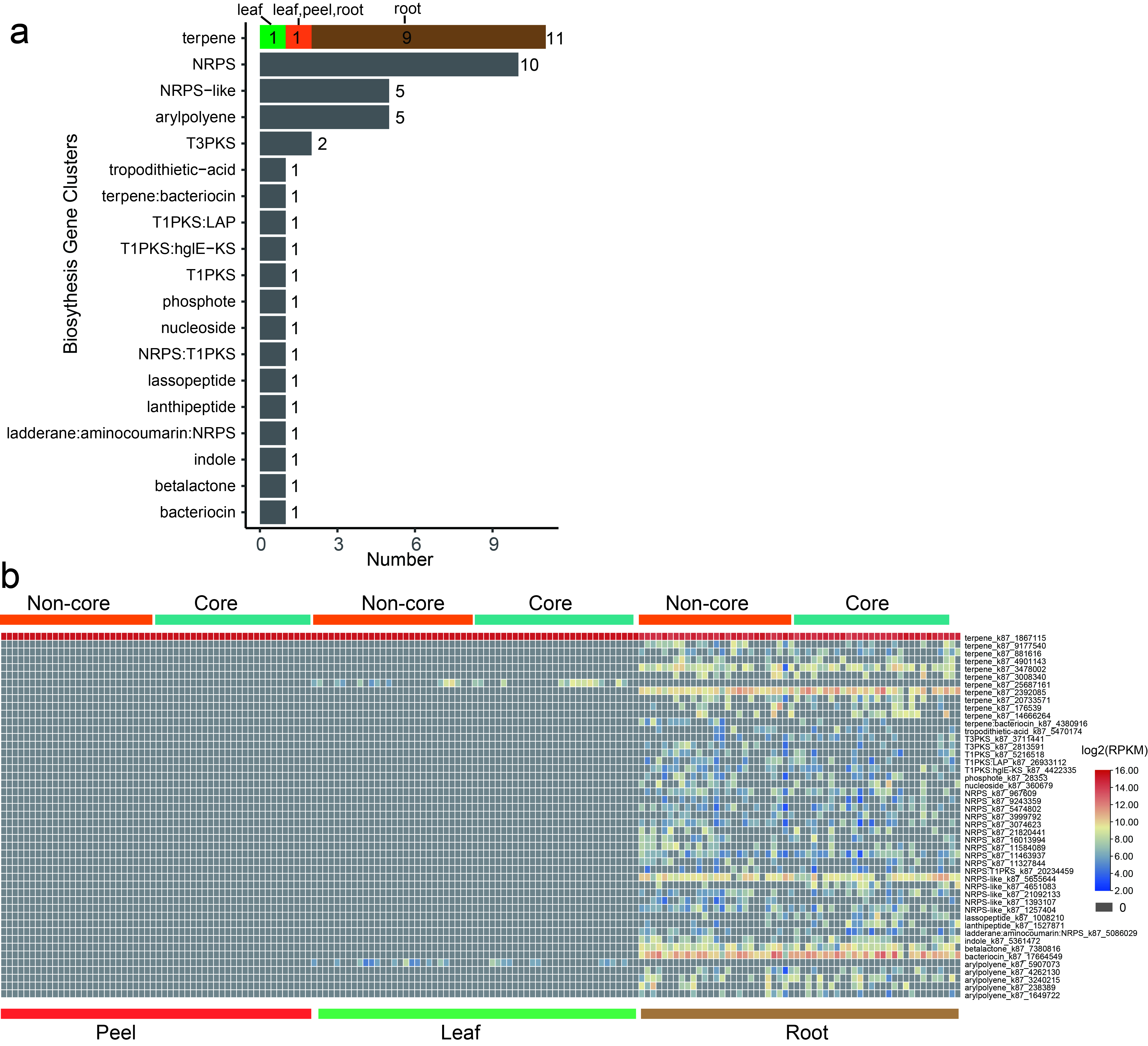

Supplement: Supplementary file 3 — Additional file 2: Figure S1. Differentially expressed genes in leaf, peel and root samples between the two regions. (a) The numbers in the figure represent the number of differentially expressed genes. (b) FPKM of 1-deoxy-D-xylulose-5-phosphate synthase (DXS) between regions. Statistical differences in peel, leaves, and roots between the two regions were evaluated by the Wilcoxon rank sum test. (c) Relative expression of DXS between regions was measured using qRT–PCR. Statistical differences in peel, leaves, and roots between the two regions were evaluated by the Wilcoxon rank sum test. Figure S2. Correlation network of transcript KOs and monoterpenes. The correlation-based network between highly expressed genes in the leaves (a) and peels (b) (nodes) and monoterpenes (triangles). Node size corresponds to the degree of each monoterpene. The thickness and colour of the edges denote the strength and significance, respectively. Solid and dashed edges indicate positive and negative correlations, respectively. Figure S3. The taxonomic composition of the rhizosphere soil microbiome at the phylum level. Only the microbial phyla with the top 10 relative abundances among bacteria (a) and archaeal phyla (b) are shown. Figure S4. Microbial composition of the root-associated microbiome and its relationship to soil chemical properties. (a) PCoA based on the genus abundance profile was performed to assess the influences of geographical location and microhabitat on microbial communities. (b) Pairwise comparisons of environmental factors are shown, with a colour gradient denoting Spearman’s correlation coefficient. Taxonomic (endophyte and metagenomes) and functional composition relationships with each environmental factor were detected by partial Mantel tests. Edge width corresponds to Mantel’s R statistic for the corresponding distance correlations, and edge colour denotes the statistical significance based on 9,999 permutations. Solid and dashed edges indicate positive and nega [file 40168_2023_1504_MOESM2_ESM.zip › Supplementary Figures/Figure S11.jpg]

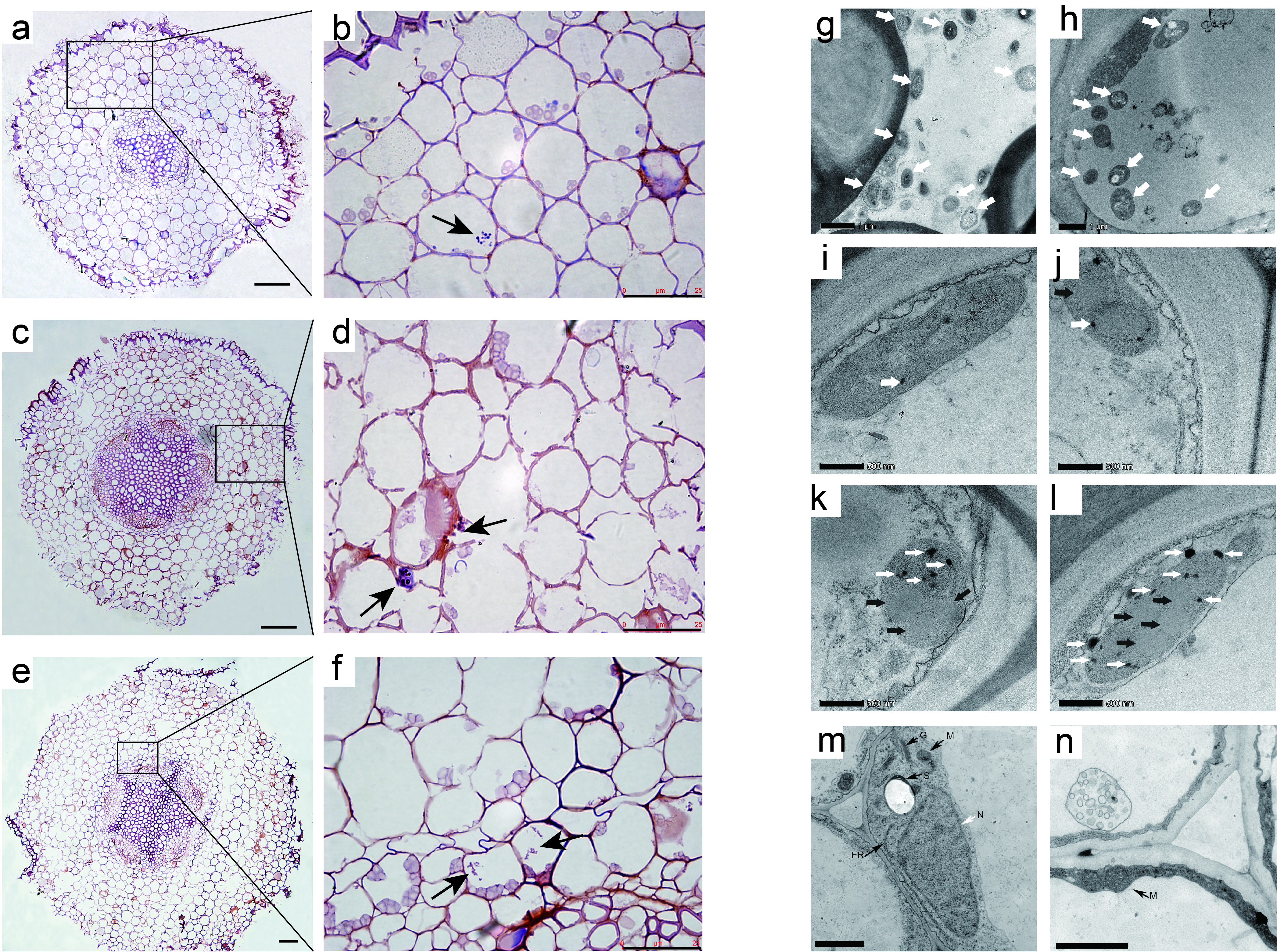

Supplement: Supplementary file 3 — Additional file 2: Figure S1. Differentially expressed genes in leaf, peel and root samples between the two regions. (a) The numbers in the figure represent the number of differentially expressed genes. (b) FPKM of 1-deoxy-D-xylulose-5-phosphate synthase (DXS) between regions. Statistical differences in peel, leaves, and roots between the two regions were evaluated by the Wilcoxon rank sum test. (c) Relative expression of DXS between regions was measured using qRT–PCR. Statistical differences in peel, leaves, and roots between the two regions were evaluated by the Wilcoxon rank sum test. Figure S2. Correlation network of transcript KOs and monoterpenes. The correlation-based network between highly expressed genes in the leaves (a) and peels (b) (nodes) and monoterpenes (triangles). Node size corresponds to the degree of each monoterpene. The thickness and colour of the edges denote the strength and significance, respectively. Solid and dashed edges indicate positive and negative correlations, respectively. Figure S3. The taxonomic composition of the rhizosphere soil microbiome at the phylum level. Only the microbial phyla with the top 10 relative abundances among bacteria (a) and archaeal phyla (b) are shown. Figure S4. Microbial composition of the root-associated microbiome and its relationship to soil chemical properties. (a) PCoA based on the genus abundance profile was performed to assess the influences of geographical location and microhabitat on microbial communities. (b) Pairwise comparisons of environmental factors are shown, with a colour gradient denoting Spearman’s correlation coefficient. Taxonomic (endophyte and metagenomes) and functional composition relationships with each environmental factor were detected by partial Mantel tests. Edge width corresponds to Mantel’s R statistic for the corresponding distance correlations, and edge colour denotes the statistical significance based on 9,999 permutations. Solid and dashed edges indicate positive and nega [file 40168_2023_1504_MOESM2_ESM.zip › Supplementary Figures/Figure S12.jpg]

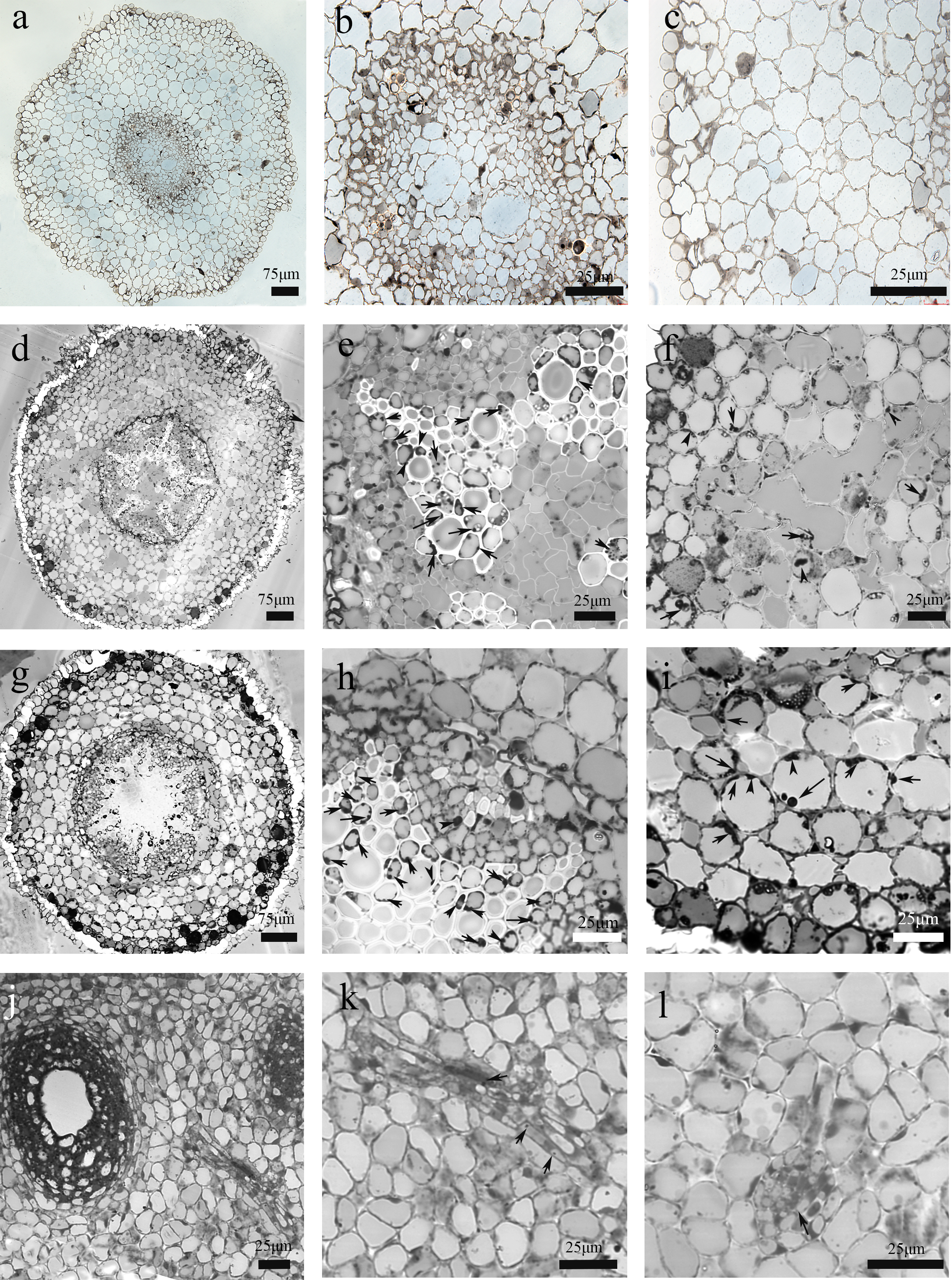

Supplement: Supplementary file 3 — Additional file 2: Figure S1. Differentially expressed genes in leaf, peel and root samples between the two regions. (a) The numbers in the figure represent the number of differentially expressed genes. (b) FPKM of 1-deoxy-D-xylulose-5-phosphate synthase (DXS) between regions. Statistical differences in peel, leaves, and roots between the two regions were evaluated by the Wilcoxon rank sum test. (c) Relative expression of DXS between regions was measured using qRT–PCR. Statistical differences in peel, leaves, and roots between the two regions were evaluated by the Wilcoxon rank sum test. Figure S2. Correlation network of transcript KOs and monoterpenes. The correlation-based network between highly expressed genes in the leaves (a) and peels (b) (nodes) and monoterpenes (triangles). Node size corresponds to the degree of each monoterpene. The thickness and colour of the edges denote the strength and significance, respectively. Solid and dashed edges indicate positive and negative correlations, respectively. Figure S3. The taxonomic composition of the rhizosphere soil microbiome at the phylum level. Only the microbial phyla with the top 10 relative abundances among bacteria (a) and archaeal phyla (b) are shown. Figure S4. Microbial composition of the root-associated microbiome and its relationship to soil chemical properties. (a) PCoA based on the genus abundance profile was performed to assess the influences of geographical location and microhabitat on microbial communities. (b) Pairwise comparisons of environmental factors are shown, with a colour gradient denoting Spearman’s correlation coefficient. Taxonomic (endophyte and metagenomes) and functional composition relationships with each environmental factor were detected by partial Mantel tests. Edge width corresponds to Mantel’s R statistic for the corresponding distance correlations, and edge colour denotes the statistical significance based on 9,999 permutations. Solid and dashed edges indicate positive and nega [file 40168_2023_1504_MOESM2_ESM.zip › Supplementary Figures/Figure S13.jpg]

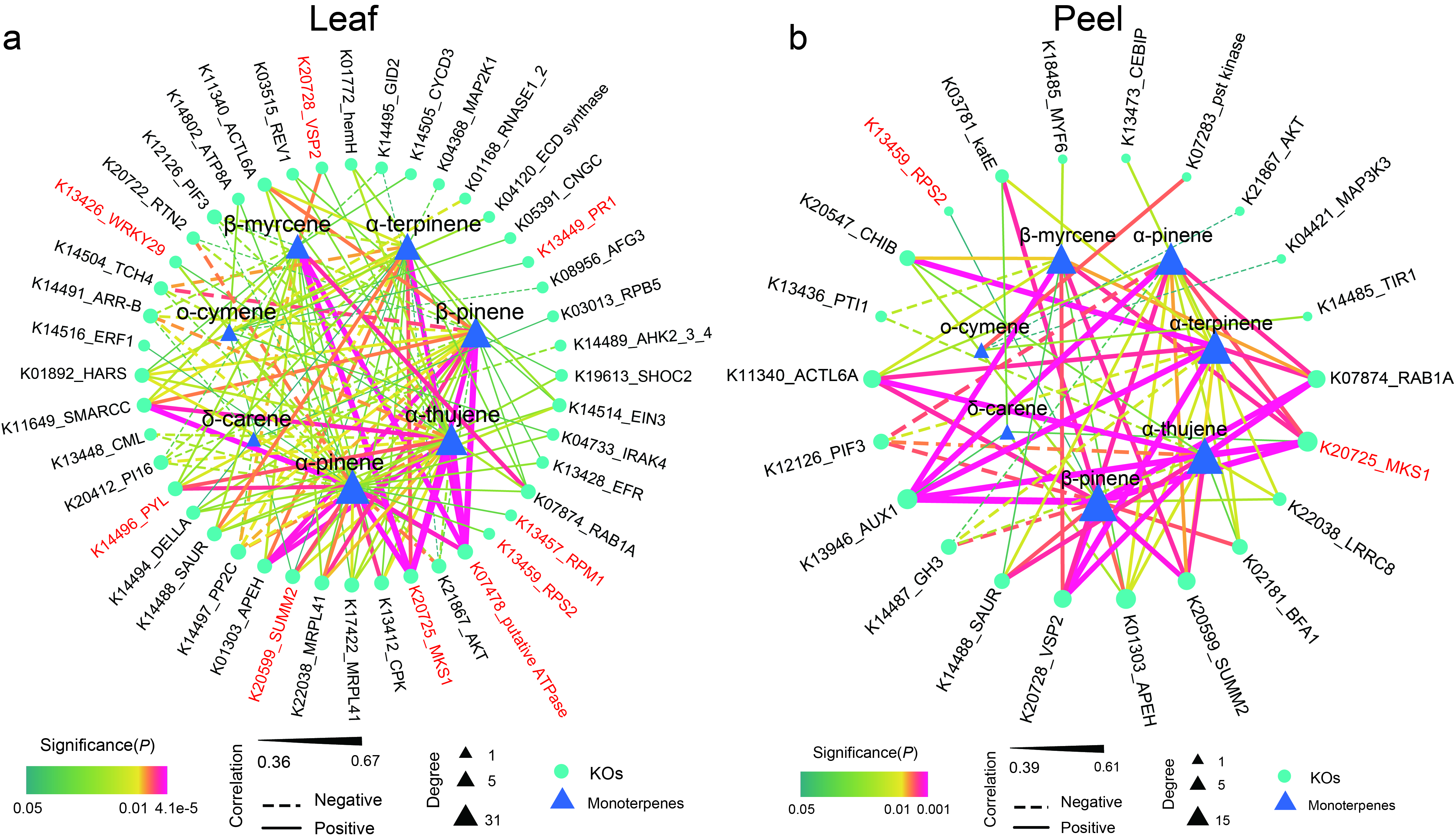

Supplement: Supplementary file 3 — Additional file 2: Figure S1. Differentially expressed genes in leaf, peel and root samples between the two regions. (a) The numbers in the figure represent the number of differentially expressed genes. (b) FPKM of 1-deoxy-D-xylulose-5-phosphate synthase (DXS) between regions. Statistical differences in peel, leaves, and roots between the two regions were evaluated by the Wilcoxon rank sum test. (c) Relative expression of DXS between regions was measured using qRT–PCR. Statistical differences in peel, leaves, and roots between the two regions were evaluated by the Wilcoxon rank sum test. Figure S2. Correlation network of transcript KOs and monoterpenes. The correlation-based network between highly expressed genes in the leaves (a) and peels (b) (nodes) and monoterpenes (triangles). Node size corresponds to the degree of each monoterpene. The thickness and colour of the edges denote the strength and significance, respectively. Solid and dashed edges indicate positive and negative correlations, respectively. Figure S3. The taxonomic composition of the rhizosphere soil microbiome at the phylum level. Only the microbial phyla with the top 10 relative abundances among bacteria (a) and archaeal phyla (b) are shown. Figure S4. Microbial composition of the root-associated microbiome and its relationship to soil chemical properties. (a) PCoA based on the genus abundance profile was performed to assess the influences of geographical location and microhabitat on microbial communities. (b) Pairwise comparisons of environmental factors are shown, with a colour gradient denoting Spearman’s correlation coefficient. Taxonomic (endophyte and metagenomes) and functional composition relationships with each environmental factor were detected by partial Mantel tests. Edge width corresponds to Mantel’s R statistic for the corresponding distance correlations, and edge colour denotes the statistical significance based on 9,999 permutations. Solid and dashed edges indicate positive and nega [file 40168_2023_1504_MOESM2_ESM.zip › Supplementary Figures/Figure S2.jpg]

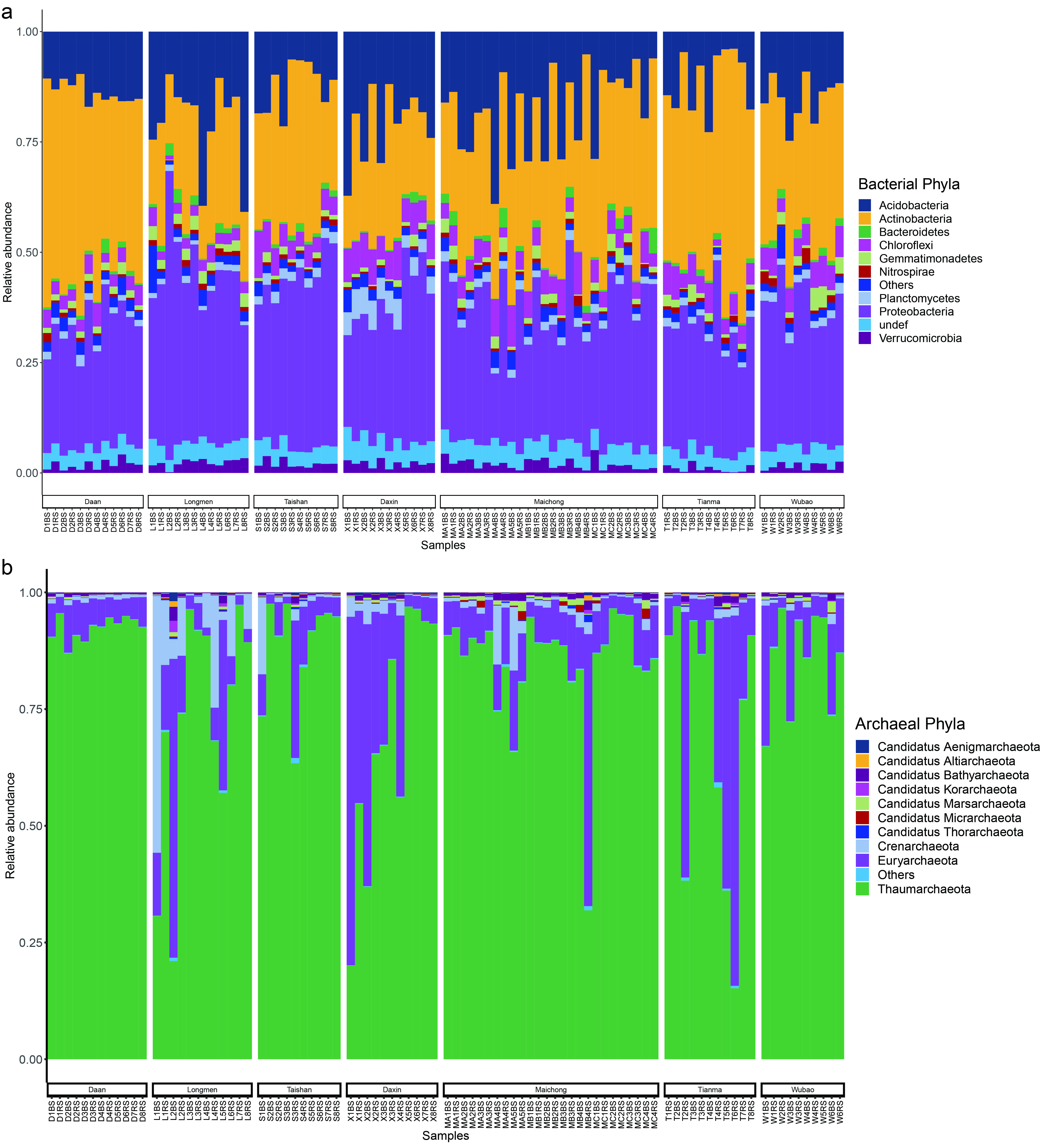

Supplement: Supplementary file 3 — Additional file 2: Figure S1. Differentially expressed genes in leaf, peel and root samples between the two regions. (a) The numbers in the figure represent the number of differentially expressed genes. (b) FPKM of 1-deoxy-D-xylulose-5-phosphate synthase (DXS) between regions. Statistical differences in peel, leaves, and roots between the two regions were evaluated by the Wilcoxon rank sum test. (c) Relative expression of DXS between regions was measured using qRT–PCR. Statistical differences in peel, leaves, and roots between the two regions were evaluated by the Wilcoxon rank sum test. Figure S2. Correlation network of transcript KOs and monoterpenes. The correlation-based network between highly expressed genes in the leaves (a) and peels (b) (nodes) and monoterpenes (triangles). Node size corresponds to the degree of each monoterpene. The thickness and colour of the edges denote the strength and significance, respectively. Solid and dashed edges indicate positive and negative correlations, respectively. Figure S3. The taxonomic composition of the rhizosphere soil microbiome at the phylum level. Only the microbial phyla with the top 10 relative abundances among bacteria (a) and archaeal phyla (b) are shown. Figure S4. Microbial composition of the root-associated microbiome and its relationship to soil chemical properties. (a) PCoA based on the genus abundance profile was performed to assess the influences of geographical location and microhabitat on microbial communities. (b) Pairwise comparisons of environmental factors are shown, with a colour gradient denoting Spearman’s correlation coefficient. Taxonomic (endophyte and metagenomes) and functional composition relationships with each environmental factor were detected by partial Mantel tests. Edge width corresponds to Mantel’s R statistic for the corresponding distance correlations, and edge colour denotes the statistical significance based on 9,999 permutations. Solid and dashed edges indicate positive and nega [file 40168_2023_1504_MOESM2_ESM.zip › Supplementary Figures/Figure S3.jpg]

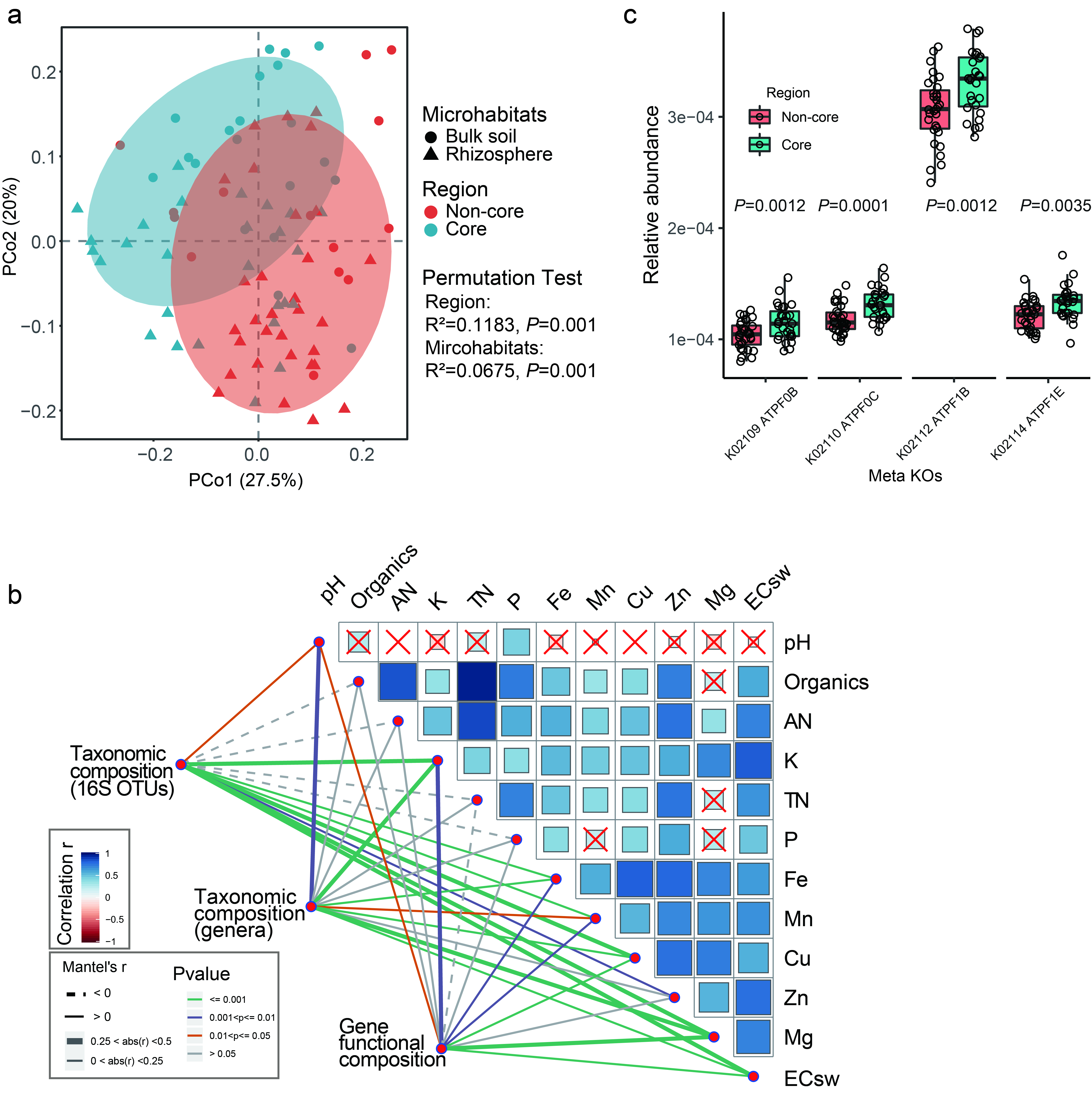

Supplement: Supplementary file 3 — Additional file 2: Figure S1. Differentially expressed genes in leaf, peel and root samples between the two regions. (a) The numbers in the figure represent the number of differentially expressed genes. (b) FPKM of 1-deoxy-D-xylulose-5-phosphate synthase (DXS) between regions. Statistical differences in peel, leaves, and roots between the two regions were evaluated by the Wilcoxon rank sum test. (c) Relative expression of DXS between regions was measured using qRT–PCR. Statistical differences in peel, leaves, and roots between the two regions were evaluated by the Wilcoxon rank sum test. Figure S2. Correlation network of transcript KOs and monoterpenes. The correlation-based network between highly expressed genes in the leaves (a) and peels (b) (nodes) and monoterpenes (triangles). Node size corresponds to the degree of each monoterpene. The thickness and colour of the edges denote the strength and significance, respectively. Solid and dashed edges indicate positive and negative correlations, respectively. Figure S3. The taxonomic composition of the rhizosphere soil microbiome at the phylum level. Only the microbial phyla with the top 10 relative abundances among bacteria (a) and archaeal phyla (b) are shown. Figure S4. Microbial composition of the root-associated microbiome and its relationship to soil chemical properties. (a) PCoA based on the genus abundance profile was performed to assess the influences of geographical location and microhabitat on microbial communities. (b) Pairwise comparisons of environmental factors are shown, with a colour gradient denoting Spearman’s correlation coefficient. Taxonomic (endophyte and metagenomes) and functional composition relationships with each environmental factor were detected by partial Mantel tests. Edge width corresponds to Mantel’s R statistic for the corresponding distance correlations, and edge colour denotes the statistical significance based on 9,999 permutations. Solid and dashed edges indicate positive and nega [file 40168_2023_1504_MOESM2_ESM.zip › Supplementary Figures/Figure S4.jpg]

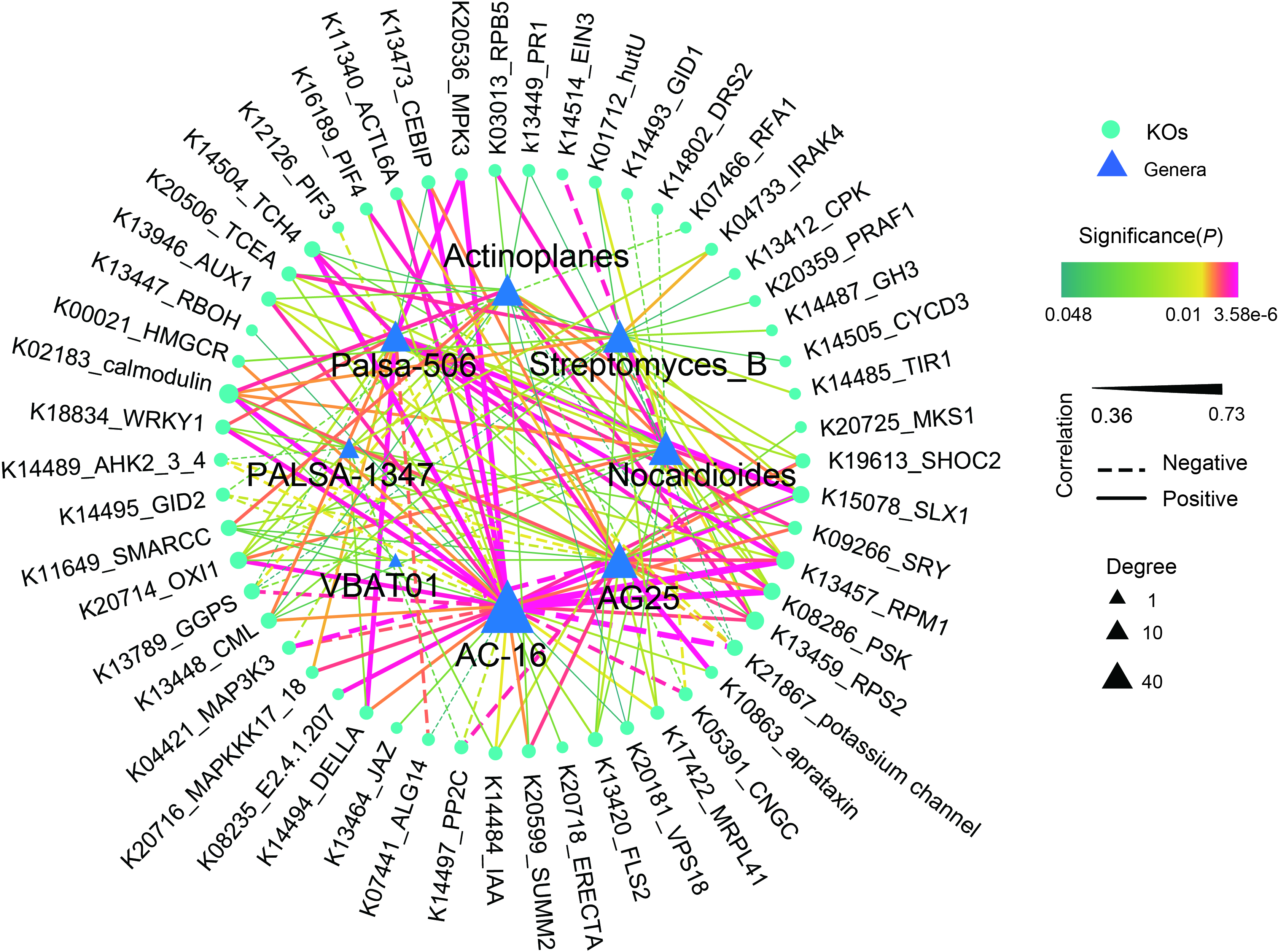

Supplement: Supplementary file 3 — Additional file 2: Figure S1. Differentially expressed genes in leaf, peel and root samples between the two regions. (a) The numbers in the figure represent the number of differentially expressed genes. (b) FPKM of 1-deoxy-D-xylulose-5-phosphate synthase (DXS) between regions. Statistical differences in peel, leaves, and roots between the two regions were evaluated by the Wilcoxon rank sum test. (c) Relative expression of DXS between regions was measured using qRT–PCR. Statistical differences in peel, leaves, and roots between the two regions were evaluated by the Wilcoxon rank sum test. Figure S2. Correlation network of transcript KOs and monoterpenes. The correlation-based network between highly expressed genes in the leaves (a) and peels (b) (nodes) and monoterpenes (triangles). Node size corresponds to the degree of each monoterpene. The thickness and colour of the edges denote the strength and significance, respectively. Solid and dashed edges indicate positive and negative correlations, respectively. Figure S3. The taxonomic composition of the rhizosphere soil microbiome at the phylum level. Only the microbial phyla with the top 10 relative abundances among bacteria (a) and archaeal phyla (b) are shown. Figure S4. Microbial composition of the root-associated microbiome and its relationship to soil chemical properties. (a) PCoA based on the genus abundance profile was performed to assess the influences of geographical location and microhabitat on microbial communities. (b) Pairwise comparisons of environmental factors are shown, with a colour gradient denoting Spearman’s correlation coefficient. Taxonomic (endophyte and metagenomes) and functional composition relationships with each environmental factor were detected by partial Mantel tests. Edge width corresponds to Mantel’s R statistic for the corresponding distance correlations, and edge colour denotes the statistical significance based on 9,999 permutations. Solid and dashed edges indicate positive and nega [file 40168_2023_1504_MOESM2_ESM.zip › Supplementary Figures/Figure S5.jpg]

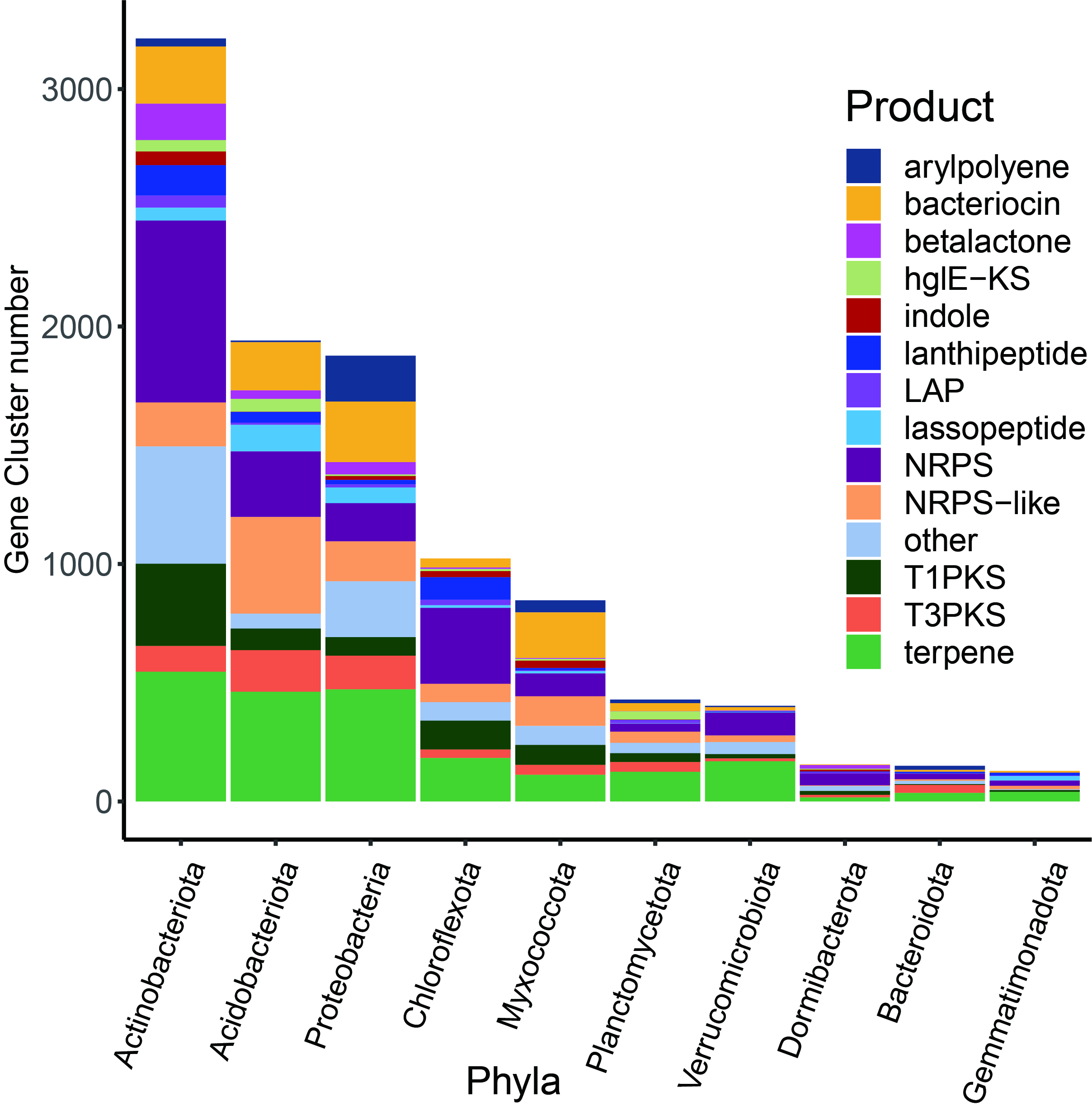

Supplement: Supplementary file 3 — Additional file 2: Figure S1. Differentially expressed genes in leaf, peel and root samples between the two regions. (a) The numbers in the figure represent the number of differentially expressed genes. (b) FPKM of 1-deoxy-D-xylulose-5-phosphate synthase (DXS) between regions. Statistical differences in peel, leaves, and roots between the two regions were evaluated by the Wilcoxon rank sum test. (c) Relative expression of DXS between regions was measured using qRT–PCR. Statistical differences in peel, leaves, and roots between the two regions were evaluated by the Wilcoxon rank sum test. Figure S2. Correlation network of transcript KOs and monoterpenes. The correlation-based network between highly expressed genes in the leaves (a) and peels (b) (nodes) and monoterpenes (triangles). Node size corresponds to the degree of each monoterpene. The thickness and colour of the edges denote the strength and significance, respectively. Solid and dashed edges indicate positive and negative correlations, respectively. Figure S3. The taxonomic composition of the rhizosphere soil microbiome at the phylum level. Only the microbial phyla with the top 10 relative abundances among bacteria (a) and archaeal phyla (b) are shown. Figure S4. Microbial composition of the root-associated microbiome and its relationship to soil chemical properties. (a) PCoA based on the genus abundance profile was performed to assess the influences of geographical location and microhabitat on microbial communities. (b) Pairwise comparisons of environmental factors are shown, with a colour gradient denoting Spearman’s correlation coefficient. Taxonomic (endophyte and metagenomes) and functional composition relationships with each environmental factor were detected by partial Mantel tests. Edge width corresponds to Mantel’s R statistic for the corresponding distance correlations, and edge colour denotes the statistical significance based on 9,999 permutations. Solid and dashed edges indicate positive and nega [file 40168_2023_1504_MOESM2_ESM.zip › Supplementary Figures/Figure S6.jpg]

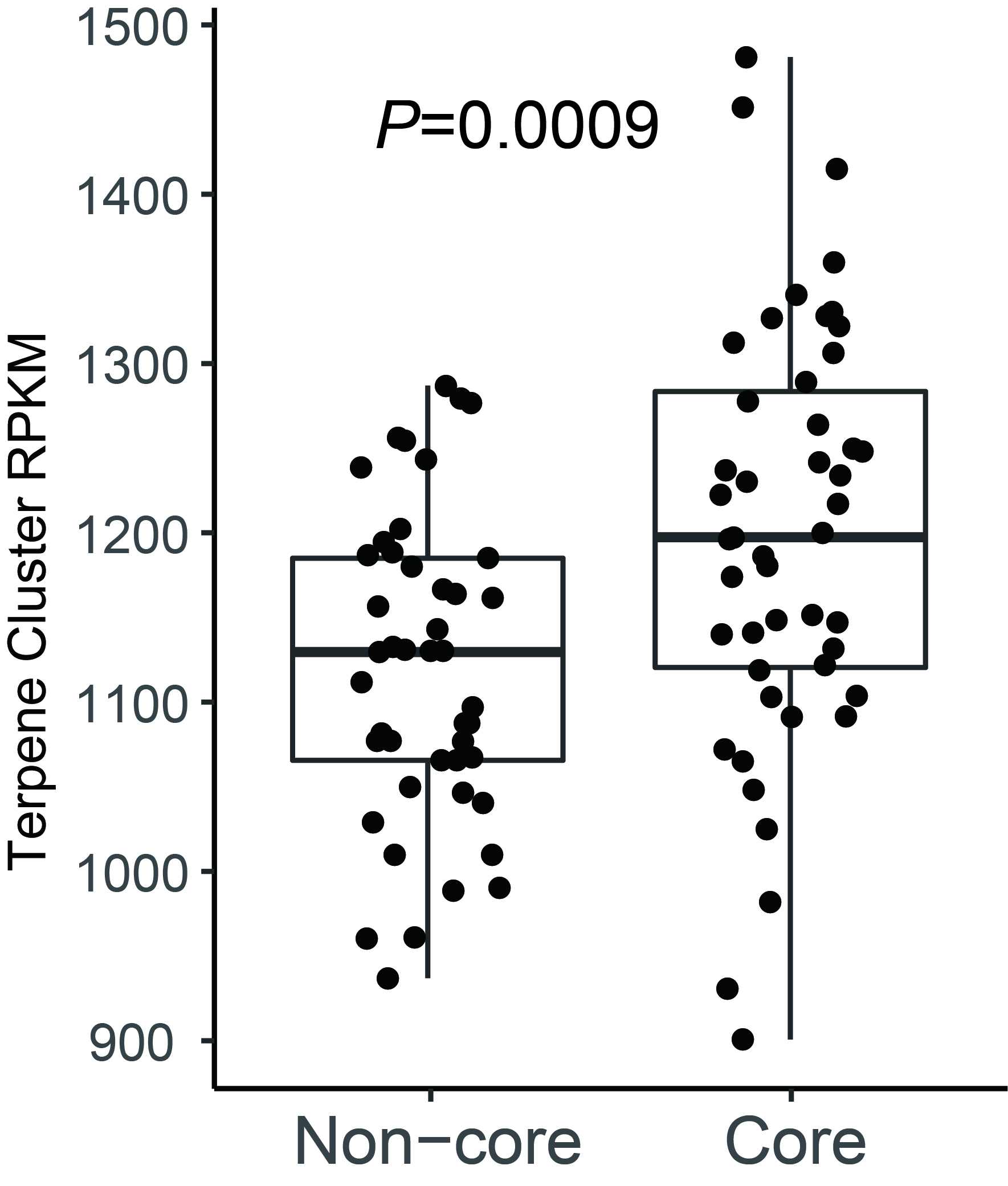

Supplement: Supplementary file 3 — Additional file 2: Figure S1. Differentially expressed genes in leaf, peel and root samples between the two regions. (a) The numbers in the figure represent the number of differentially expressed genes. (b) FPKM of 1-deoxy-D-xylulose-5-phosphate synthase (DXS) between regions. Statistical differences in peel, leaves, and roots between the two regions were evaluated by the Wilcoxon rank sum test. (c) Relative expression of DXS between regions was measured using qRT–PCR. Statistical differences in peel, leaves, and roots between the two regions were evaluated by the Wilcoxon rank sum test. Figure S2. Correlation network of transcript KOs and monoterpenes. The correlation-based network between highly expressed genes in the leaves (a) and peels (b) (nodes) and monoterpenes (triangles). Node size corresponds to the degree of each monoterpene. The thickness and colour of the edges denote the strength and significance, respectively. Solid and dashed edges indicate positive and negative correlations, respectively. Figure S3. The taxonomic composition of the rhizosphere soil microbiome at the phylum level. Only the microbial phyla with the top 10 relative abundances among bacteria (a) and archaeal phyla (b) are shown. Figure S4. Microbial composition of the root-associated microbiome and its relationship to soil chemical properties. (a) PCoA based on the genus abundance profile was performed to assess the influences of geographical location and microhabitat on microbial communities. (b) Pairwise comparisons of environmental factors are shown, with a colour gradient denoting Spearman’s correlation coefficient. Taxonomic (endophyte and metagenomes) and functional composition relationships with each environmental factor were detected by partial Mantel tests. Edge width corresponds to Mantel’s R statistic for the corresponding distance correlations, and edge colour denotes the statistical significance based on 9,999 permutations. Solid and dashed edges indicate positive and nega [file 40168_2023_1504_MOESM2_ESM.zip › Supplementary Figures/Figure S7.jpg]

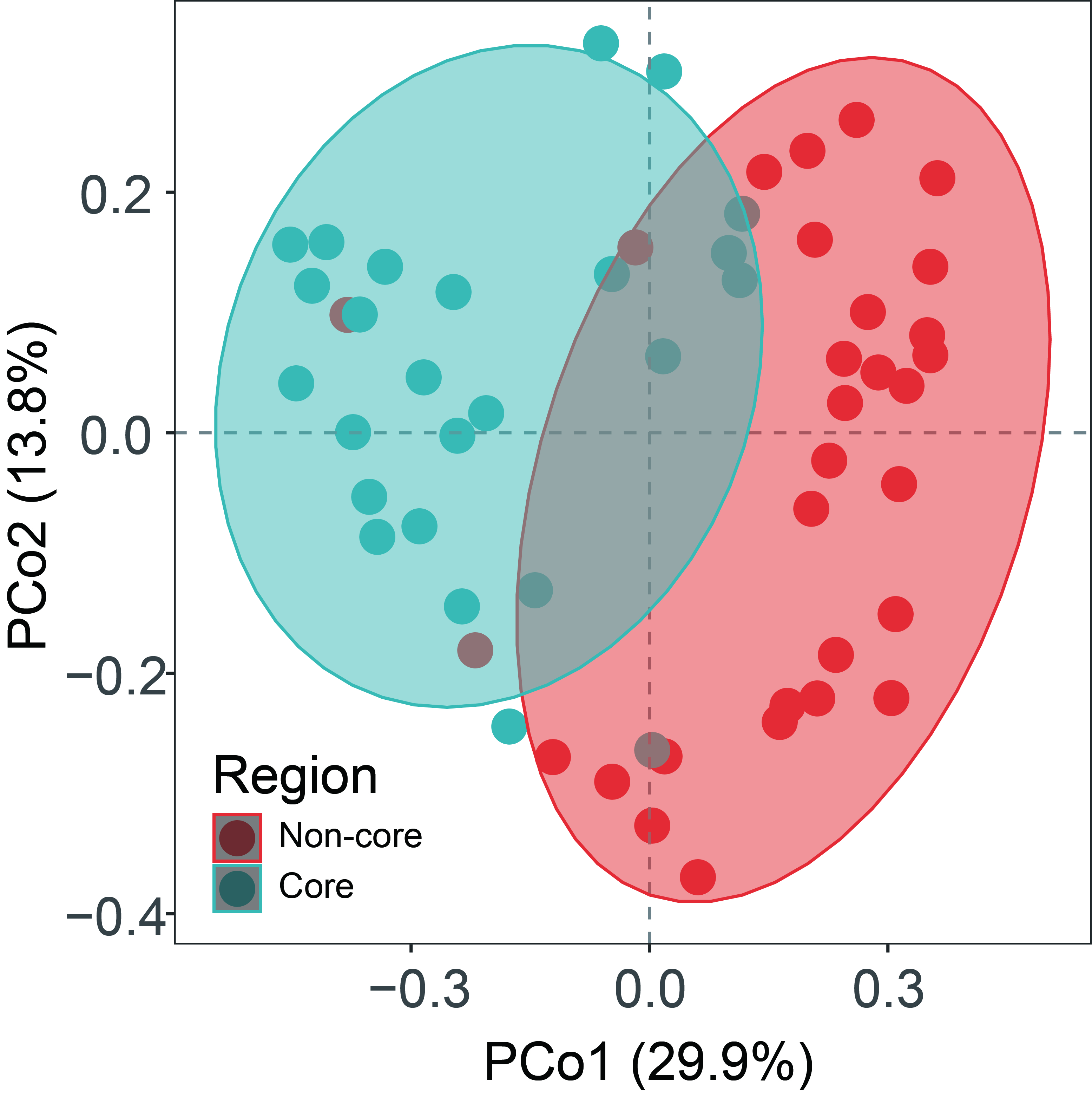

Supplement: Supplementary file 3 — Additional file 2: Figure S1. Differentially expressed genes in leaf, peel and root samples between the two regions. (a) The numbers in the figure represent the number of differentially expressed genes. (b) FPKM of 1-deoxy-D-xylulose-5-phosphate synthase (DXS) between regions. Statistical differences in peel, leaves, and roots between the two regions were evaluated by the Wilcoxon rank sum test. (c) Relative expression of DXS between regions was measured using qRT–PCR. Statistical differences in peel, leaves, and roots between the two regions were evaluated by the Wilcoxon rank sum test. Figure S2. Correlation network of transcript KOs and monoterpenes. The correlation-based network between highly expressed genes in the leaves (a) and peels (b) (nodes) and monoterpenes (triangles). Node size corresponds to the degree of each monoterpene. The thickness and colour of the edges denote the strength and significance, respectively. Solid and dashed edges indicate positive and negative correlations, respectively. Figure S3. The taxonomic composition of the rhizosphere soil microbiome at the phylum level. Only the microbial phyla with the top 10 relative abundances among bacteria (a) and archaeal phyla (b) are shown. Figure S4. Microbial composition of the root-associated microbiome and its relationship to soil chemical properties. (a) PCoA based on the genus abundance profile was performed to assess the influences of geographical location and microhabitat on microbial communities. (b) Pairwise comparisons of environmental factors are shown, with a colour gradient denoting Spearman’s correlation coefficient. Taxonomic (endophyte and metagenomes) and functional composition relationships with each environmental factor were detected by partial Mantel tests. Edge width corresponds to Mantel’s R statistic for the corresponding distance correlations, and edge colour denotes the statistical significance based on 9,999 permutations. Solid and dashed edges indicate positive and nega [file 40168_2023_1504_MOESM2_ESM.zip › Supplementary Figures/Figure S8..jpg]

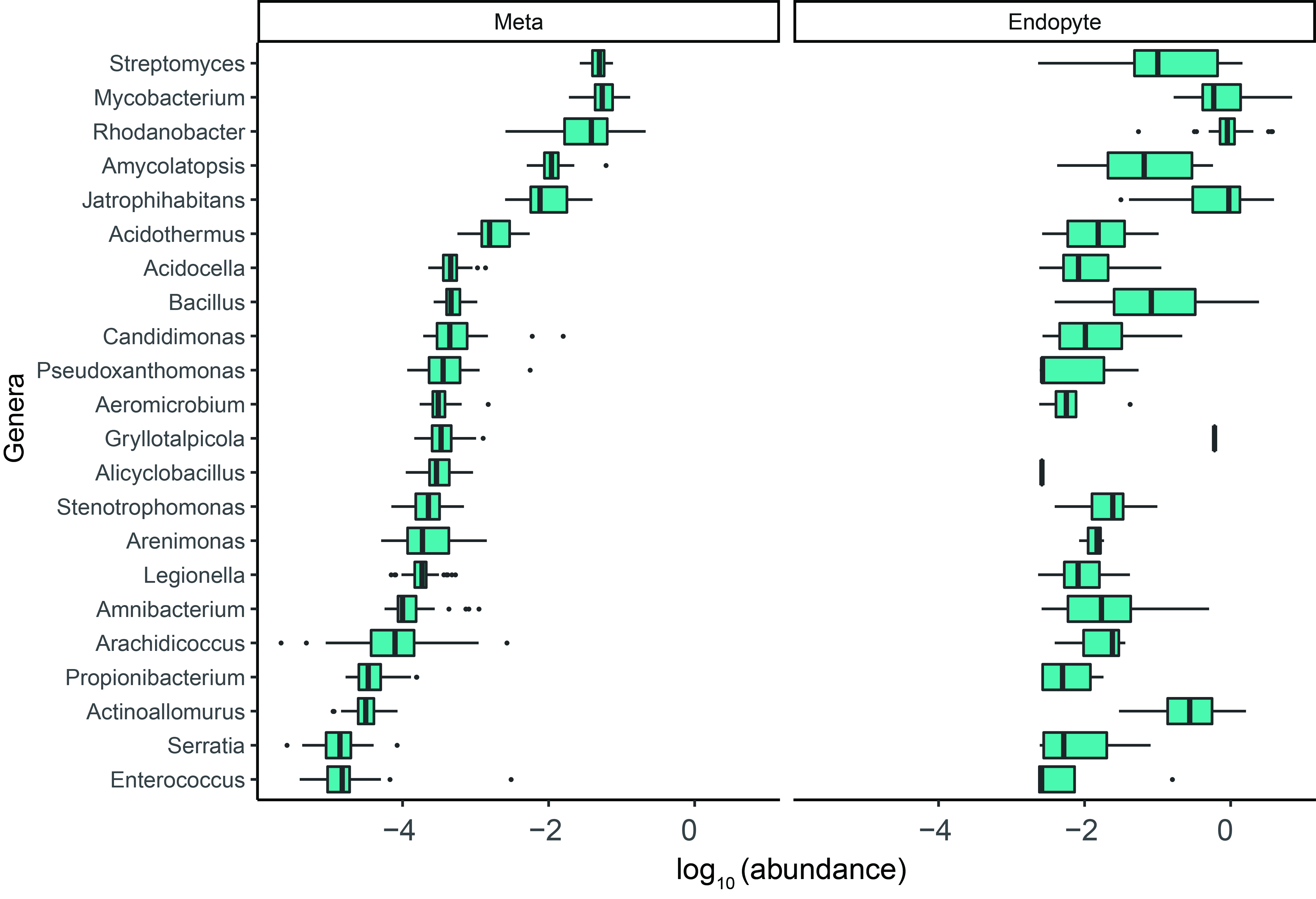

Supplement: Supplementary file 3 — Additional file 2: Figure S1. Differentially expressed genes in leaf, peel and root samples between the two regions. (a) The numbers in the figure represent the number of differentially expressed genes. (b) FPKM of 1-deoxy-D-xylulose-5-phosphate synthase (DXS) between regions. Statistical differences in peel, leaves, and roots between the two regions were evaluated by the Wilcoxon rank sum test. (c) Relative expression of DXS between regions was measured using qRT–PCR. Statistical differences in peel, leaves, and roots between the two regions were evaluated by the Wilcoxon rank sum test. Figure S2. Correlation network of transcript KOs and monoterpenes. The correlation-based network between highly expressed genes in the leaves (a) and peels (b) (nodes) and monoterpenes (triangles). Node size corresponds to the degree of each monoterpene. The thickness and colour of the edges denote the strength and significance, respectively. Solid and dashed edges indicate positive and negative correlations, respectively. Figure S3. The taxonomic composition of the rhizosphere soil microbiome at the phylum level. Only the microbial phyla with the top 10 relative abundances among bacteria (a) and archaeal phyla (b) are shown. Figure S4. Microbial composition of the root-associated microbiome and its relationship to soil chemical properties. (a) PCoA based on the genus abundance profile was performed to assess the influences of geographical location and microhabitat on microbial communities. (b) Pairwise comparisons of environmental factors are shown, with a colour gradient denoting Spearman’s correlation coefficient. Taxonomic (endophyte and metagenomes) and functional composition relationships with each environmental factor were detected by partial Mantel tests. Edge width corresponds to Mantel’s R statistic for the corresponding distance correlations, and edge colour denotes the statistical significance based on 9,999 permutations. Solid and dashed edges indicate positive and nega [file 40168_2023_1504_MOESM2_ESM.zip › Supplementary Figures/Figure S9.jpg]
